# Studies on the Changes of Pharmacokinetics Behaviors of Phytochemicals and the Influence on Endogenous Metabolites After the Combination of Radix Bupleuri and Radix Paeoniae Alba Based on Multi-Component Pharmacokinetics and Metabolomics

**DOI:** 10.3389/fphar.2021.630970

**Published:** 2021-03-08

**Authors:** Congcong Chen, Qicai Yin, Junshen Tian, Xiaoxia Gao, Xuemei Qin, Guanhua Du, Yuzhi Zhou

**Affiliations:** ^1^ Modern Research Center for Traditional Chinese Medicine of Shanxi University, Taiyuan, China; ^2^ College of Chemistry and Chemical Engineering, Shanxi University, Taiyuan, China; ^3^ Institute of Materia Medica, Chinese Academy of Medical Sciences and Peking Union Medical College, Beijing, China

**Keywords:** Radix Bupleuri-Radix Paeoniae Alba herb pair, multi-component pharmacokinetics, metabolomics, correlation analysis, herb-herb compatibility

## Abstract

Radix Bupleuri-Radix Paeoniae Alba (RB-RPA) is a classic herb pair, which is commonly used to treat depression by soothing “liver qi stagnation” in the clinic. However, little is yet known concerning the combination mechanism of Radix Bupleuri (RB) and Radix Paeoniae Alba (RPA), their bioactive forms *in vivo* and the regulatory effects on the organism. The present study aimed to elucidate the changes in multi-component pharmacokinetics (PK) behavior after the combination of RB and RPA by a high-resolution full-scan mode of UPLC-HRMS method (a total of 38 components PK profiles were obtained, of which 23 components come from RB and 15 components come from RPA). Moreover, the metabolomics approach was used to analyze the dynamic response of endogenous metabolites intervened by RB-RPA, and the correlation between concentration-time curves of 38 components from RB-RPA and the dynamic response profiles of endogenous metabolites was characterized by Pearson correlation analysis. The results demonstrated that the combination of RB and RPA could significantly improve the bioavailability of five components in RB, and six components in RPA. Besides, metabolomics results indicated that a total of 21 endogenous metabolites exhibited time-dependent changes in response to the RB-RPA administration, of which 12 endogenous metabolites were significantly increased, and nine endogenous metabolites were significantly decreased. Furthermore, correlation analysis results indicated that the components with significantly improved bioavailability after combination such as saikogenin F, saikogenin G, albiflorin, methyl gallate, paeonimetabolin II were significantly positively correlated with picolinic acid, a metabolite with neuroprotective effect; saikogenin F, saikogenin G were significantly positively correlated with itaconic acid, a endogenous metabolite with anti-inflammatory activity; and albiflorin, paeonimetabolin II were significantly positively correlated with α-linolenic acid, a metabolite with strong protective actions on brain functions. These results indicated that the combination of RB and RPA can enhance each other’s neuroprotective and anti-inflammatory activities. In this study, A novel and efficient strategy has been developed to analyze the influence of the combination of RB and RPA *in vivo* behaviors by combining multi-component pharmacokinetics with metabolomics, which was contributed to clarifying the scientific connotation of herb–herb compatibility.

**Figure:**
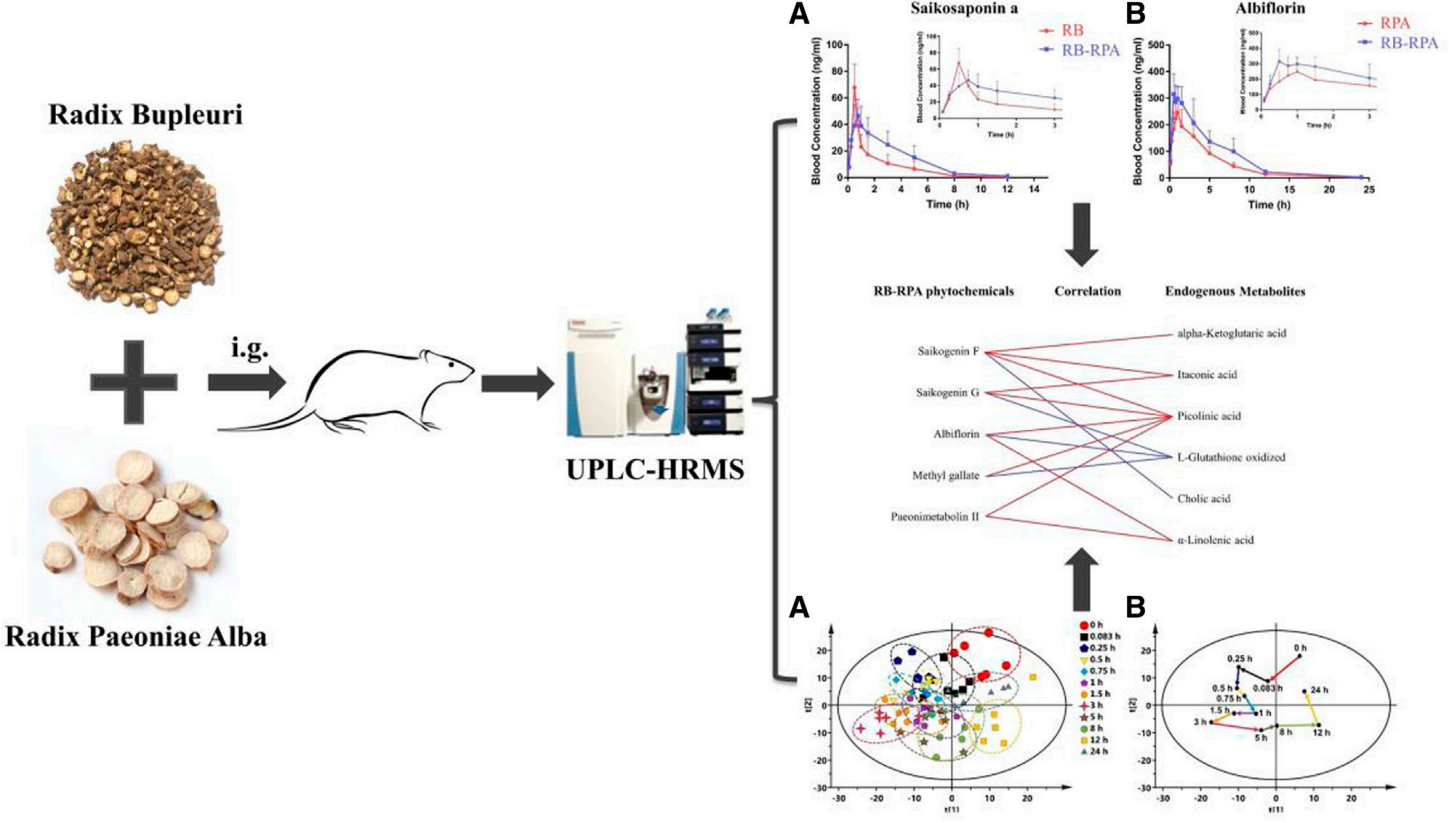
**GRAPHICAL ABSTRACT |** A novel and efficient strategy have been developed to analyze the influence of compatibility of RB and RPA *in vivo* behaviors by combining multi-component pharmacokinetics with metabolomics.

## Introduction

RB is the dried root of the umbelliferous plant *Bupleurum chinense* DC. It has been widely used in Asia for thousands of years due to its pharmaceutical effects on antipyretic, analgesic, anti-inflammatory and anti-depressant (Ashour and Wink, 2011). The saponins such as saikosaponin A, saikosaponin D were the main active components of antidepressant effect (Tian et al., 2016). RPA is derived from the dried roots of *Paeonia lactiflora* Pall. In “Shennong’s Classic of Material Medical,” the efficacy of RPA was described in detail. Modern pharmacological studies have found that RPA has a variety of biological activities, such as analgesia, anti-inflammatory, immune-enhancing and anti-depression effects (Xu et al., 2008). Paeoniflorin and albiflorin were the main active ingredients in the antidepressant effect of RPA (Qiu et al., 2013). RB-RPA was a common herb pair, which was regarded as the core drug pair in Xiaoyaosan, Sinisan and Chaihu-Shugan San which all were classic prescriptions for treating depression, and exerted antidepressant effect by relieving “liver qi stagnation.” Herb–herb compatibility is a common form of TCM, it can achieve an optimal effect by obtaining synergy or reducing possible adverse reactions (Zhao et al., 2010). Modern pharmacological studies have shown that the analgesic, anti-inflammatory, and antidepressant effect was significantly improved after the combination of RB and RPA (Wang et al., 2016; Li et al., 2021). However, the scientific connotation of RB and RPA combination and the potential theoretical basis of increasing therapeutic effect after combination were unclear. Currently, pharmacokinetic research has become a reliable way to elucidate the synergistic mechanism of herb–herb compatibility, as it can reflect the dynamic changes of the pharmacodynamic substances before and after compatibility (Wang et al., 2012; Zhou et al., 2017).

Recently, we have also analyzed the chemical components in rat plasma after oral administration of RB-RPA herb pair, a total of 55 components were detected in rat plasma, of which 16 were prototype components and 39 were metabolites of prototype components (Yin et al., 2019). The analysis of the chemical profile of RB-RPA in rat plasma makes it possible to conduct a comprehensive PK study. In this work, we employed the Thermo-Fisher Dionex UltiMate 3000 UHPLC-Q Exactive Orbitrap-MS system, with a high-resolution and high-throughput platform to conduct the PK study of 38 marker compounds (due to the low concentration of the other 17 components, the time points that could be detected were less than six, their PK curves were not available), and the mass spectrum information and extracted ion ranges of the 38 components were listed in Table 1. The method was based on a high-resolution full-scan mode, to acquire a comprehensive profile of all ionized components in rat plasma, and previous studies have confirmed the reliability of this analytical method in PK studies (Wang et al., 2019). So, based on the above research, the impact on pharmacokinetic parameters before and after the combination of RB and RPA was analyzed by multi-component pharmacokinetics research method.

**TABLE 1:** The prototype components and their metabolites characterized by UPLC/MS/MS after oral administration of RB-RPA herb pair in rat plasma, and the extracted ion ranges of 38 components.

No.	Name	*t*_R_/min	Formula	Ion mode	m/z	Source or parent	Extracted ion ranges
P1	Desbenzoylpaeoniflorin	0.972	C_16_H_24_O_10_	[M + HCOO]^−^	421.13408	RPA	421.13405–421.13410
P2	Methyl gallate	3.473	C_8_H_8_O_5_	[M-H]^−^	183.02991	RPA	183.02988–183.02993
P3	Oxypaeoniflorin	4.695	C_23_H_28_O_12_	[M-H]^−^	495.15087	RPA	495.15084–495.15090
P4	Albiflorin	6.658	C_23_H_28_O_11_	[M + HCOO]^−^	525.16021	RPA	525.16019–525.16024
P5	Paeoniflorin	7.693	C_23_H_28_O_11_	[M + HCOO]^−^	525.16029	RPA	525.16026–525.16032
P6	Saikosaponin C	13.856	C_48_H_78_O_17_	[M + HCOO]^−^	971.52109	RB	971.52107–971.52112
P7	Saikosaponin A	15.069	C_42_H_68_O_13_	[M + HCOO]^−^	825.45521	RB	825.45518–825.45523
P8	Saikosaponin B_2_	15.516	C_42_H_68_O_13_	[M + HCOO]^−^	825.45520	RB	825.45518–825.45523
P9	Acetyl-saikosaponin A	16.183	C_44_H_70_O_14_	[M + HCOO]^−^	867.47362	RB	867.47360–867.47370
P10	Saikosaponin D	16.525	C_42_H_68_O_13_	[M + HCOO]^−^	825.45521	RB	825.45518–825.45523
P11	Acetyl-saikosaponin B_2_	16.615	C_44_H_70_O_14_	[M + HCOO]^−^	867.47364	RB	867.47360–867.47370
P12	Acetyl-saikosaponin D	17.583	C_44_H_70_O_14_	[M + HCOO]^−^	867.47368	RB	867.47360–867.47370
M1	Paeonimetabolin I	9.674	C_10_H_14_O_4_	[M-H]^−^	197.08117	Paeoniflorin	197.08115–197.08123
M2	Paeonimetabolin II	11.311	C_10_H_16_O_4_	[M-H]^-^	199.09755	Paeoniflorin	199.09753–199.09758
M3	Paeonimetabolin I glucuronide isomer	6.033	C_16_H_22_O_10_	[M-H]^−^	373.11411	Paeoniflorin	373.11408–373.11414
M4	Methylgallic acid glucuronide	6.957	C_14_H_16_O_11_	[M-H]^−^	359.06197	Methyl gallate	359.06195–359.06199
M5	Methylgallic acid sulfate	2.968	C_8_H_8_O_8_S	[M-H]^−^	262.98679	Methyl gallate	262.98675–262.98682
M6	3,4-di-*O*-methyl gallic acid sulfate	10.353	C_9_H_10_O_8_S	[M-H]^−^	277.00231	Methyl gallate	277.00228–277.00233
M7	Pyrogallol glucuronide	1.284	C_12_H_14_O_9_	[M-H]^−^	301.05658	Methyl gallate	301.05656–301.05662
M8	Pyrogallol sulfate	6.745	C_6_H_6_O_6_S	[M-H]^−^	204.98135	Methyl gallate	204.98133–204.98138
M9	Methylpyrogallol sulfate	2.082	C_7_H_8_O_6_S	[M-H]^−^	218.99695	Methyl gallate	218.99692–218.99698
M10	Methylpyrogallol glucuronide	1.049	C_13_H_16_O_9_	[M-H]^−^	315.07209	Methyl gallate	315.07206–315.07213
M11	Prosaikogenin F	14.972	C_36_H_57_O_8_	[M + HCOO]^−^	662.40253	Saikosaponin A	662.40247–662.40257
M12	Saikogenin F	13.123	C_30_H_48_O_4_	[M-H_2_O + H]^+^	455.35191	Saikosaponin A	455.35188–455.35198
M13	Hydroxy-saikogenin F	13.905	C_30_H_48_O_5_	[M-H_2_O + H]^+^	471.34678	Saikosaponin A	471.34674–471.34692
M14	Dihydroxyl-dehydrogenation-saikogenin F	13.021	C_30_H_46_O_6_	[M-H_2_O + H]^+^	485.32603	Saikosaponin A	485.32600–485.32606
M15	Hydroxyl-dehydrogenation-saikogenin F	16.142	C_30_H_46_O_5_	[M-H_2_O + H]^+^	469.33111	Saikosaponin A	469.33108–469.33118
M16	Dihydroxyl-saikogenin F	13.648	C_30_H_48_O_6_	[M-H_2_O + H]^+^	487.34168	Saikosaponin A	487.34164–487.34172
M17	Trihydroxyl-dehydrogenation-saikogenin F	13.336	C_30_H_46_O_7_	[M-H_2_O + H]^+^	501.32095	Saikosaponin A	501.32090–501.32099
M18	Saikogenin E	16.086	C_30_H_48_O_3_	[M-H_2_O + H]^+^	439.35692	Saikosaponin C	439.35686–439.35696
M19	Hydroxy-saikogenin E	17.372	C_30_H_48_O_4_	[M-H_2_O + H]^+^	455.35182	Saikosaponin C	455.35178–455.35186
M20	Dihydroxyl-dehydrogenation-saikogenin E	16.321	C_30_H_46_O_5_	[M-H_2_O + H]^+^	469.33115	Saikosaponin C	469.33108–469.33118
M21	Hydroxyl-dehydrogenation-saikogenin E	16.894	C_30_H_46_O_4_	[M-H_2_O + H]^+^	453.33621	Saikosaponin C	453.33618–453.33625
M22	Dihydroxyl-saikogenin E	14.757	C_30_H_48_O_5_	[M-H_2_O + H]^+^	471.34669	Saikosaponin C	471.34666–471.34676
M23	Prosaikogenin G	17.669	C_36_H_57_O_8_	[M + HCOO]^-^	662.40251	Saikosaponin D	662.40247–662.40257
M24	Saikogenin G	16.516	C_30_H_48_O_4_	[M-H_2_O + H]^+^	455.35196	Saikosaponin D	455.35188–455.35198
M25	Prosaikogenin D	15.947	C_36_H_57_O_8_	[M + HCOO]^-^	662.40249	Saikosaponin B_2_	662.40247–662.40257
M26	Saikogenin D	14.544	C_30_H_48_O_4_	[M-H_2_O + H]^+^	455.35193	Saikosaponin B_2_	455.35188–455.35198

P: prototype components absorbed into the plasma; M: metabolites of prototype components; RB: Radix Bupleuri; RPA: Radix Paeoniae Alba.

Distinct from chemical drugs, the large number and wide concentration range of compounds were present in TCM. Besides, the vast number of compounds were ingested would have a series of regulatory effects in the body (including endogenous metabolites that were significantly regulated in response to the intake of herbal medicines compounds), multi-compounds interact with multi-targets to achieve a maximal therapeutic effect and could exert a holistic treatment to multi-targets diseases such as depression (Xue and Roy, 2003). Therefore, establishing the evidence-based pharmacokinetics (PK) and pharmacodynamics (PD) research methods for multicomponent TCM was still a difficult issue. It was worth noting that many of today’s major diseases (such as diabetes, hyperuricemia, and depression) have a strong metabolic foundation or a definite metabolic cause (Wishart, 2016). Besides, the nutraceutical intervention of multicomponent herbal medicines was regarded as a process in which the plant metabolome interacts with the body metabolome (Xie et al., 2018). Therefore, endogenous metabolites as an indicator of PD become a reliable method to solve this difficult problem (Zhang et al., 2019). At the same time, revealing the dynamic response and interactions between herbal phytochemistry and endogenous metabolites was provided a new opportunity to clarify the holistic and synergistic mechanisms of TCM (Xie et al., 2012). In this study, metabolomics technology was applied to analyze the dynamic response of endogenous metabolites after oral RB-RPA. At the same time, PK-PD correlation analysis was used to comprehensively analyze the effect of “plant metabolome” on “body metabolome” by integrating pharmacokinetics and metabonomics technology.

## Materials and Methods

### Chemicals and Reagents

HPLC grade acetonitrile, methanol, and LC-MS grade formic acid were obtained from Thermo-Fisher Scientific Inc. (United States). Purification of deionized water using the Milli-Q system (Millipore, Billerica, MA, United States). The Chinese Herbal Slices of Radix Bupleuri and Radix Paeoniae Alba were purchased from Anguo Qiao Chinese herbal sliced medicine Co., Ltd. and the batch number were 1710436111 and 1708255131 respectively. Moreover, Traditional Chinese medicines Radix Bupleuri and Radix Paeoniae Alba were authenticated by Prof. Xue-Mei Qin of Shanxi University, which confirmed that Radix Bupleuri is the dried root of the umbelliferous plant *Bupleurum chinense* DC and Radix Paeoniae Alba is derived from the dried roots of *Paeonia lactiflora* Pall. Voucher specimens of Radix Bupleuri and Radix Paeoniae Alba were deposited in the Modern Research Center for Traditional Chinese Medicine of Shanxi University, labeled as YZ-2018–0403001 (Radix Paeoniae Alba) and YZ-2018–0403002 (Radix Bupleuri), respectively. Saikosaponin A (batch number BWB50206), saikosaponin D (batch number BWB50210), saikosaponin C (batch number BWB50209), saikosaponin B_2_ (batch number BWB50208) and methyl gallate (batch number BWB50638) were purchased from Chengdu Ruifensi Biological Technology Co., Ltd. (Sichuan, China). Paeoniflorin (batch number Y0001856), albiflorin (batch number ASB-00001513–005), oxypaeoniflorin (batch number BWB50094) and glycyrrhizin (IS; batch number 14110717) were purchased from the Chinese National Institute of Pharmaceutical and Biological Products (Beijing, China). The purities of all standards were at least 98%, and all other organic reagents were of analytical grade.

### Preparation of Herb Extracts

As described in previous reports (Chen et al., 2020; Li et al., 2021), Radix Bupleuri (3 kg) or Radix Paeoniae Alba (3 kg) were soaked in 70% ethanol (2.4 L) for 1.5 h before extraction. Then Radix Bupleuri or Radix Paeoniae Alba were extracted twice with 70% ethanol under reflux, each time for 1.5 h. The extracts were filtrated and concentrated in vacuo and lyophilized into powders (15.15% yield for Radix Bupleuri, and 11.78% yield for Radix Paeoniae Alba), and then stored at 4°C until use and UPLC-MS analysis.

Besides, to assure the quality of Radix Bupleuri and Radix Paeoniae Alba, the chemical fingerprinting was analyzed by HPLC. For Radix Bupleuri, the saikosaponin A, saikosaponin D, saikosaponin B_2,_ and saikosaponin C were identified as chemical markers for quality monitoring; For Radix Paeoniae Alba, the albiflorin, paeoniflorin, oxypaeoniflorin, and methyl gallate were identified as chemical markers for quality monitoring. The representative HPLC was shown in Supplementary Figure S1 and the content of the eight constituents in herb extracts was shown in Supplementary Table S1.

### Preparation of Standards, Calibration Standards, and QC Samples

Individual stock solutions (1.00 mg mL^−1^) of saikosaponin A, saikosaponin D, saikosaponin C, saikosaponin B_2_, paeoniflorin, albiflorin, oxypaeoniflorin and methyl gallate were prepared by accurately weighing the required amounts into volumetric flasks and dissolving in methanol. The individual stock solutions were serially diluted with methanol and then mixed to provide working standard solutions of the desired concentrations. The Internal standard (IS) stock solutions of glycyrrhizin (1.00 ug mL^−1^) were also prepared in methanol, and then diluted with methanol to the desired concentrations of 300 ng mL^−1^.

Calibration standard solutions were prepared by spiking 50 μL of a mixed standard solution with 150 μL blank rat plasma to give a desired concentrations: saikosaponin D, saikosaponin C, saikosaponin B_2_, and oxypaeoniflorin at 0.1, 0.2, 0.5, 1.0, 5.0, 10.0, 20.0, 50.0, 250.0 ng mL^−1^; saikosaponin A and methyl gallate at 0.2, 0.4, 1.0, 2.0, 10.0, 20.0, 40.0, 100.0, 500.0 ng mL^−1^; albiflorin at 0.5, 1.0, 2.5, 5.0, 25.0, 50.0, 100.0, 250.0, 1250.0 ng mL^−1^; paeoniflorin at 2.0, 4.0, 10.0, 20.0, 100.0, 200.0, 400.0, 1000.0, 5000.0 ng mL^−1^.

For method validation, QC samples were prepared using three concentration levels of the standard solution in the same manner, with the desired concentrations of saikosaponin D, saikosaponin C, saikosaponin B_2_, and oxypaeoniflorin at 0.2, 5.0, 50.0 ng mL^−1^; saikosaponin A and methyl gallate at 0.4, 10.0, 100.0 ng mL^−1^; albiflorin at 1.0, 25.0, 250.0 ng mL^−1^; paeoniflorin at 4.0, 100.0, 1000.0 ng mL^−1^.

### Plasma Sample Pretreatment

150 μL of plasma was mixed with 50 μL of IS solution and 300 μL methanol-water (1:1, v/v). The above mixtures were vortexed for 2 min, ultrasonicated for 5 min, and then centrifuged at 4°C/13,000 rpm for 15 min. The supernatant was separated and evaporated to dryness with a SCIENTZ-50F vacuum centrifugal concentrator (Scientz Biotechnology Co., Ltd., Ningbo, China). The dry extracts were reconstituted in 150 ul methanol-water (1:5, v/v), vortexed for 2 min, and centrifuged at 4°C/13,000 rpm for 10 min. Finally, transferred 100 μL to autosampler vials for UPLC-MS analysis.

### Animal Handing and Sampling

Male Sprague-Dawley rats, weighing 220 ± 20 g (aged 8 weeks), were provided by the Beijing Vital Laboratory Animal Co., Ltd. (Beijing, China, No. SCXK2018-0011). All of the rats were adapted to the novel experimental environment for 7 days (room temperature 22 ± 2°C, 55 ± 5% relative humidity and 12 h light-dark cycle); All rats were free to access the water and food until 12 h before the experiment. The animal study was reviewed and approved by the Experimental Animal Ethical Committee of Modern Research Center for Traditional Chinese Medicine, Shanxi University (animal ethic approval number: SXULL2018018), and all experimental procedures were carried out in accordance with the NIH Guide for the Care and Use of Laboratory Animals. After one week of adaptation, twenty-one rats were divided into three groups randomly, with seven rats in each group: 1) RPA group, were oral administration of Radix Paeoniae Alba extract (45 g-herb/kg); 2) RB group, were oral administration of Radix Bupleuri extract (45 g-herb/kg); 3) RB-RPA group, were oral administration of powder mixture of Radix Bupleuri and Radix Paeoniae Alba extracts (containing 45 g-herb/kg Radix Bupleuri and 45 g-herb/kg Radix Paeoniae Alba). The medicinal powders of all groups were dissolved in distilled water at concentrations of 0.53 g/ml for RPA, 0.68 g/ml for RB and 1.21 g/ml for RB-RPA. Each group received intragastric administration with a volume of 10 ml/kg (rat body weight). The dosage of Radix Bupleuri and Radix Paeoniae Alba is equivalent to a 3-fold clinical dosage of component herbs in Xiaoyao San (Chen et al., 2020). The blood samples (0.3 ml) were collected from the ophthalmic venous plexus into heparinized tubes before oral administration and subsequently at 0.083, 0.25, 0.5, 0.75, 1, 1.5, 3, 5, 8, 12, and 24 h after dosing. All rats were free to access the water during the experiment. The blood samples at each time point were collected from seven rats. The blood samples were centrifuged at 4,000 rpm for 10 min and frozen at −80°C until analysis.

### UPLC-HRMS Analysis for Herb Extracts and Plasma Sample

Using a Thermo-Fisher Dionex UltiMate 3000 UHPLC system coupled with a Q Exactive Orbitrap-MS (Thermo-Fisher, United States) and Xcalibur workstation (Thermo-Fisher Scientific Inc.,Waltham, MA, United States) to acquire UPLC-HRMS raw data. Chromatographic separation of herb extracts and plasma samples was performed on an Acquity UPLC HSS T3 column (2.1 mm × 100 mm, 1.8 μm) maintained at 37°C. The flow rate was 0.2 ml/min and the injection volume was 5 μL. The mobile phase consisted of (solvent A) 0.1% formic acid in water (v/v) and (solvent B) 0.1% formic acid in acetonitrile (v/v), the gradient elution conditions for herb extracts and plasma samples were operated under the following program: 0∼5.5 min, 5% B; 5.5∼9.5 min, 5∼15% B; 9.5∼13 min, 15∼35% B; 13∼17 min, 35∼60% B; 17∼20 min, 60∼90% B; 20∼22 min, 90% B; 22∼23 min, 90∼5%B; 23∼25 min, 5% B. Mass spectrometry detection conditions were set as follows: the MS data were acquired under positive and negative ionization modes via heated electrospray ionization (HESI) source. The scan mode was Full Scan and the scan range was set 50–1000 m/z; heater temperature, 300°C; capillary temperature, 330°C; spray voltage, 3.5 kV (positive mode) and 2.6 kV (negative mode); sheath gas velocity, 35 arb; auxiliary gas flow, 10 arb. The Q Exactive Orbitrap-MS has a fast positive and negative ion switching function, which could switch between positive-negative ion modes during the analysis of the same sample. So, we applied the method of switching ion modes in the detection process to quantify all analytes better. Glycyrrhizin was selected as the internal standard because it could be detected in both positive and negative ion modes.

### Pharmacokinetics Analysis

UPLC/MS/MS calibration and quantitation data were processed with Xcalibur workstation (Thermo-Fisher Scientific Inc., Waltham, MA, United States). For the eight components with standard reference, calculating their concentrations from the standard curves. For the other 30 components without standard reference, their concentrations were analyzed following the regression equations of homologous compounds (Qiao et al., 2012): paeoniflorin for P1 and M1 to M3; methylgallate for M4 to M10; saikosaponin A for P9 and M11 to M17; saikosaponin C for M18 to M22; saikosaponin D for P12 and M23 to M24; saikosaponin B_2_ for P11 and M25 to M26. For the PK studies, the maximum concentration (*C*_max_), time of maximum plasma concentration (*t*_max_), terminal elimination half-life (*t*_1/2_), areas under the concentration-time curve (AUC_0-*t*_ and AUC_0-∞_) of each compound were analyzed by a non-compartmental analysis using Drug And Statistics Version 3.0 (DAS 3.0) software (Mathematical Pharmacology Committee, Chinese Pharmacological Society, China).

### Analysis of Radix Bupleuri-Radix Paeoniae Alba Herb Pair-Induced Endogenous Metabolites Variations

The UPLC-HRMS raw data were imported to Compound Discoverer 3.0 (Thermo Fisher, United States) for matching and aligning peak data. The parameters were set as follows: mass tolerance, 5 ppm; RT tolerance, 0.05 min; S/N threshold, 10; intensity tolerance, 30%; assignment threshold, 60; mass range, 50–1000 Da. The peak area data of all metabolites obtained from Compound Discoverer 3.0 was normalized by IS (glycyrrhizin) in Microsoft Excel 2013.

To analyze the RB-RPA herb pair-induced endogenous metabolites variations, the acquired data at each time point from Compound Discoverer 3.0 (removal of 55 RB-RPA herb pair metabolites previously identified in rats plasma (Yin et al., 2019)) were imported into SIMCA-P software (version 16.0, Umetrics, Sweden) for multivariate statistical analysis, such as the principal components analysis (PCA), partial least-squares discriminant analysis (PLS-DA) and orthogonal partial least-squares discriminant analysis (OPLS-DA). The altered endogenous metabolites were screened according to the VIP-value of S-plot (VIP >1) and T-test (*p* < 0.05). The selected metabolites of LC-MS analysis were identified based on the molecular formula, accurate m/z values, MS/MS fragments, and online databases including KEGG (http://www.kegg.jp), m/z cloud (https://www.mzcloud.org/), PubChem (https://pubchem.ncbi.nlm.nih.gov/), HMDB (http://www.hmdb.ca), Lipid Maps (http://www. lipidmaps.org), and Massbank (http://www.massbank.jp). Meanwhile, based on the investigation of metabonomics and the semi-quantification, the mean value of each altered endogenous metabolite at each time point, representing the average response, was calculated to analyze the relationship of the dynamic response for endogenous metabolites along with the time course.

### Correlation analysis of 38 Radix Bupleuri-Radix Paeoniae Alba Herb Pair Phytochemicals in Rat Plasma and Altered Endogenous Metabolites

Pearson correlation analysis was further applied to find the high linear relationship of phytochemicals and altered endogenous metabolites. To analyze the relationship of the dynamic response for endogenous metabolites along with the time course, the mean value of each endogenous metabolite was calculated at each time point (0 h → 0.083 h → 0.25 h → 0.5 h → 0.75 h → 1 h → 1.5 h → 3 h → 5 h → 8 h → 12 h → 24 h). A new metabolite vector with 12 mean values calculated at 12 different time points, representing the average response was constructed. Pearson correlation analysis to study the correlation between two new metabolite vectors (representing RB-RPA herb pair phytochemicals and altered endogenous metabolites, respectively). The |r| ≥ 0.8 and *p* <0.05 represented that there was a high correlation between RB-RPA herb pair phytochemicals and altered endogenous metabolites.

### Statistical Analyses

All data were expressed as the mean  ± standard deviation (SD). Data were statistically analyzed by SPSS 18.0 software (SPSS Inc., United States) and SIMCA-P 16.0 software (Umetrics, Sweden). PCA and PLS-DA were used to explore altered endogenous metabolites. Statistical analyses from two groups were analyzed using a two-tailed unpaired t-test, and statistical analyses from more groups were analyzed using one-way ANOVA. The *p* <0.05 were considered statistically significant.

## Result and Discussion

### Comparison of the Content of Eight Compounds Between Single Extracts and a Mixture of Radix Bupleuri and Radix Paeoniae Alba Extracts

The content of eight compounds in single (RB or RPA) extracts and mixture of RB and RPA extracts were analyzed by UPLC-HRMS. The base peak chromatograms of herb extracts and mixed standards (saikosaponin A, saikosaponin D, saikosaponin C, saikosaponin B_2_, paeoniflorin, albiflorin, oxypaeoniflorin, methyl gallate, and the internal standard) were shown in Supplementary Figure S1. The content of the eight constituents in herb extracts was shown in Supplementary Table S1. The statistical results showed that the content of eight compounds had no statistical difference between single (RB or RPA) extracts and mixture extracts. These results suggested that mixing process of RB and RPA extracts didn’t affect the content changed of each component in RB or RPA extracts. Previous studies have shown that co-decoction of RB and RPA *in vitro* can significantly increase the content of paeoniflorin and galloylpaeoniflorin in the RPA extract, and significantly reduce the content of saikosaponin A in the RB extract (He et al., 2018). Therefore, in order to avoid the content changes of prototype compounds caused by co-decocting *in vitro*, we used mixture of RB and RPA extracts to analyze the impact on pharmacokinetic parameters before and after the combination of RB and RPA.

### Validation of the UPLC–MS/MS *in vivo* Analysis Method

#### Specificity

The specificity was investigated by comparing extracted-ion chromatograms (XICs) of the blank plasma sample, spiked plasma sample, and a plasma sample after oral administration of RB, RPA, and RB-RPA. As shown in Supplementary Figure S2, there were no significant endogenous substances interference peak was observed.

#### Calibration Curve and LLOQ

The calibration curves, linear ranges, correlation coefficients (r), and LLOQ of the eight analytes were represented in Supplementary Table S2. The coefficient of correlation (r^2^) values greater than 0.995 and the LLOQs varied from 0.1–2.0 ng/ml for all analytes. This suggested that the calibration curves of these analytes showed good linearity within a certain concentration range in rat plasmas.

#### Matrix Effect and Extraction Recovery

The results of the matrix effect and extraction recoveries of the eight analytes were listed in Supplementary Table S3. The extraction recoveries of the eight analytes in rat plasmas at three different concentrations were in range of 81.32–106.83%, and the matrix effects of the analytes were in range of 79.61–111.53%, indicating that there was no significant ion suppression/enhancement in this bioanalytical method.

#### Precision and Accuracy

Precision and accuracy were evaluated by analyzing QC samples at three different concentrations (low, medium, and high) in six replicates on the same day (intra-day) and on three consecutive days (inter-day), respectively. As shown in Supplementary Table S4, the intra- and inter-day precisions values (RSD) ranged from 1.82 to 9.11% and from 2.21 to 11.48%, respectively, and the accuracy values (RE) ranged from −8.11 to 8.12%. The results suggest that this method is feasible.

#### Stability

The stability of the eight analytes during the sample processing and storage procedures was assessed by analyzing five replicates for QC samples at three different concentrations. The results were summarized in Supplementary Table S5, which showed that the eight analytes in plasma were all stable at room temperature for 24 h, at autosampler (4°C) for 24 h, three freeze-thaw cycles and 30 days storage at −20°C with values of RE (%) in the range −8.92 to 6.35%.

### Pharmacokinetics

#### Determination of Plasma Concentration of 38 Compounds from “Plant Metabolome”

**FIGURE 1:**
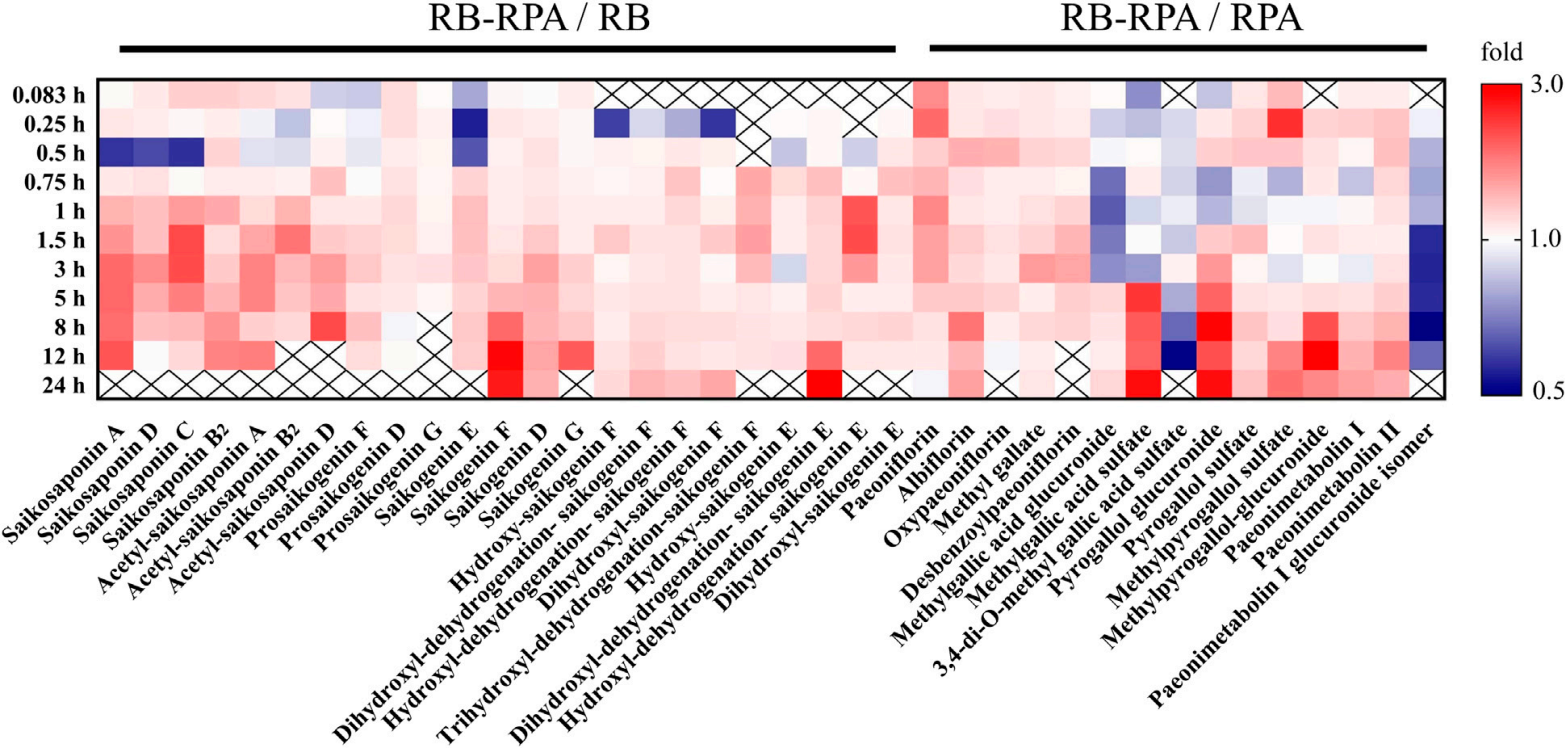
Comparison of plasma concentrations to reveal PK interactions. The average plasma concentrations (*n* = 7) between RB-RPA and RB or RPA were compared in heatmap. They were referred to as “X/Y,” where X is the Compatibility group and Y is RB or RPA single group. Red color indicates that the ratio is greater than 1, and the blue color indicates that the ratio is less than 1, see color bar scale. “×” means the data was not detected at this time points.

The validated UPLC-HRMS method was applied to simultaneously determine the plasma concentrations of 38 compounds after oral administration of RB-RPA herb pair in rats, including 12 prototype compounds and 26 metabolized products (Table 1). The peak areas of the 38 compounds were extracted from the extracted-ion chromatograms using a mass extraction window centered on the theoretical m/z, which was attributed to the high-resolution and high-throughput acquisition of HRMS. By comparing the plasma concentrations to reveal PK parameters of the 38 compounds before and after combination, we found that the combination of RB and RPA significantly changed the plasma concentrations of 38 compounds compared with the single herbal group (Figure 1).

#### Comparison of Pharmacokinetics of 23 Compounds from RB Before and After Compatibility

**FIGURE 2:**
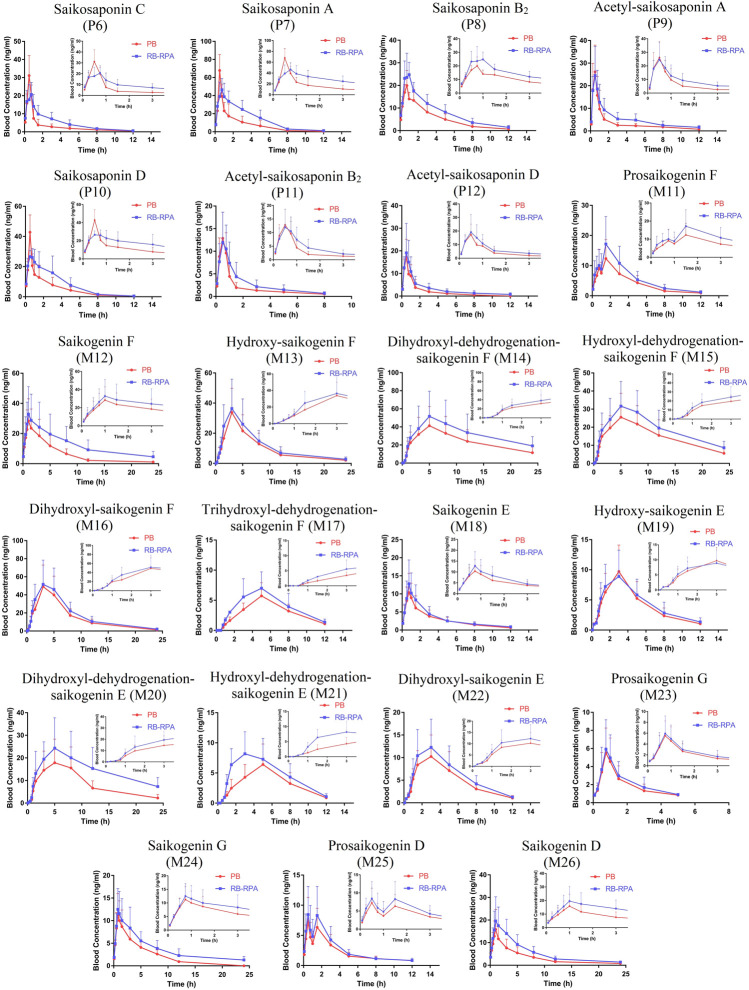
Plasma concentration-time profiles (mean ± SD, *n* = 7) of 23 compounds from RB after oral administration of the RB (single extract) and RB-RPA (RB and RPA compatibility). The compounds represented by the numbers in the figure are consistent with the compounds represented by the numbers in Table 1.

**FIGURE 3:**
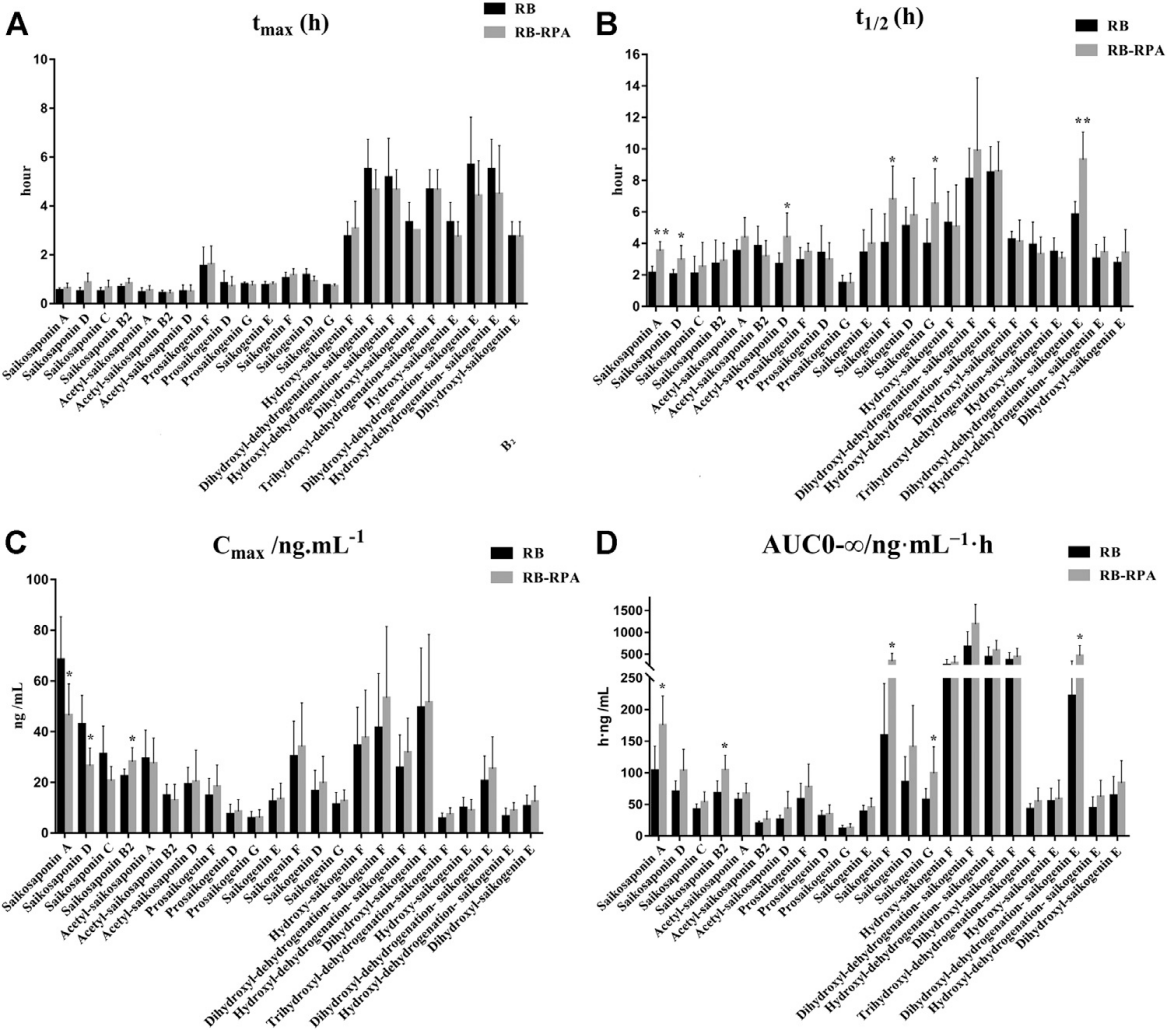
Main pharmacokinetic parameters of 23 compounds from RB in rat plasma after oral administration of the single extract group (RB) and the compatibility group (RB-RPA). **(A)** *t*_max_ (h); **(B)** *t*_1/2_ (h); **(C)** *C*_max_ (ng mL^−1^); **(D)** AUC_0-∞_ (ng mL^−1^ h). All data were expressed as mean ± SD, (*n* = 7). *p < 0.05, **p < 0.01 compared with the RB group.

The time–concentration curves of 23 compounds from RB before and after combination were shown in Figure 2. Furthermore, to clearly observe differences among the 23 compounds from RB before and after combination, the main PK parameters were calculated using a non-compartment model in DAS 3.0 software, and the results were listed in Supplementary Table S6. By comparing the PK parameters (*C*_max_, *t*_max_, *t*_1/2_, and AUC_0-∞_) of 23 compounds between the RB-RPA group and the RB groups, the results showed that the combination of RB and RPA could impact the pharmacokinetic behaviors of 23 compounds from RB (Figure 3). For saikosaponin A and saikosaponin D, the PK profile of administration of RB alone was similar to a previous report (Xu et al., 2012). When saikosaponin A and saikosaponin D were administered in RB-RPA herbs, the *C*_max_ of saikosaponin A and saikosaponin D were decreased remarkably: from 68.37 ± 16.95 to 46.47 ± 12.41, and from 42.84 ± 11.53 to 26.57 ± 6.99 ng mL^−1^, respectively (Figure 3C, Supplementary Table S6). In contrast, the *t*_1/2_ of saikosaponin A and saikosaponin D were longer in the RB-RPA group than the single herbal group, which indicated that combination can extend the residence time of saikosaponin A and saikosaponin D in system circulation (Figure 3B). As a result, the AUC_0-∞_ of saikosaponin A was significantly improved from 103.55 ± 38.59 to 175.55 ± 45.92 ng mL^−1^ h after combination (in RB-RBA group), and the AUC_0-∞_ of saikosaponin D was improved from 69.92 ± 17.38 to 103.56 ± 33.67 ng mL^−1^ h (Figure 3D). Besides, as for acetyl-saikosaponin D, prosaikogenin F, prosaikogenin G, and dihydroxyl-dehydrogenation-saikogenin E, the *t*_1/2_ of these compounds were significantly increased after combination, which indicated that combination can extend the residence time of these compounds in system circulation (Figure 3B). As a result, the AUC_0-∞_ of prosaikogenin F, prosaikogenin G, and dihydroxyl-dehydrogenation-saikogenin E were significantly increased after combination (Figure 3D). As for saikosaponin b_2_, when saikosaponin b_2_ were administered in RB-RPA herbs, the *C*_max_ increased remarkably: 22.33 ± 2.99 to 28.19 ± 5.50 ng mL^−1^, which indicated that combination can promote the absorption of saikosaponin b_2_. As a result, the AUC_0-∞_ of saikosaponin b_2_ was significantly increased after the combination. However, there was no significant effect on the *t*_max_ of 23 compounds from RB before and after combination (Figure 3A). These results indicated that the combination of RB and RPA played a critical role in improving the bioavailability of five components (saikosaponin A, saikosaponin B_2,_ prosaikogenin F, prosaikogenin G, dihydroxyl-dehydrogenation-saikogenin E) in RB.

#### Comparison of Pharmacokinetics of 15 Compounds from RPA Before and After Combination

**FIGURE 4:**
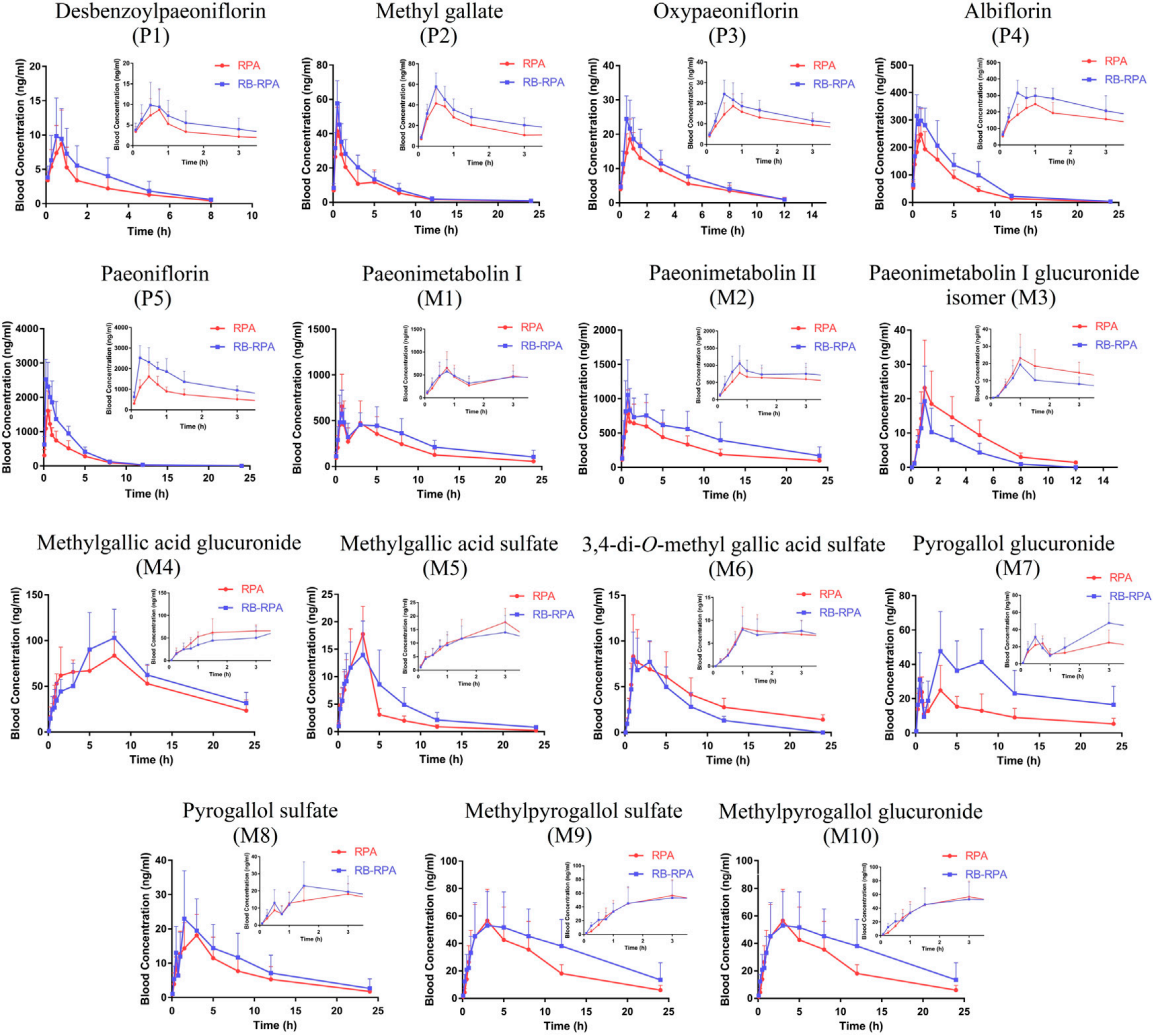
Plasma concentration-time profiles (mean ± SD, *n* = 7) of 15 compounds from RPA after oral administration of the RPA (single extract) and RB-RPA (RB and RPA compatibility). The compounds represented by the numbers in the figure are consistent with the compounds represented by the numbers in Table 1.

**FIGURE 5:**
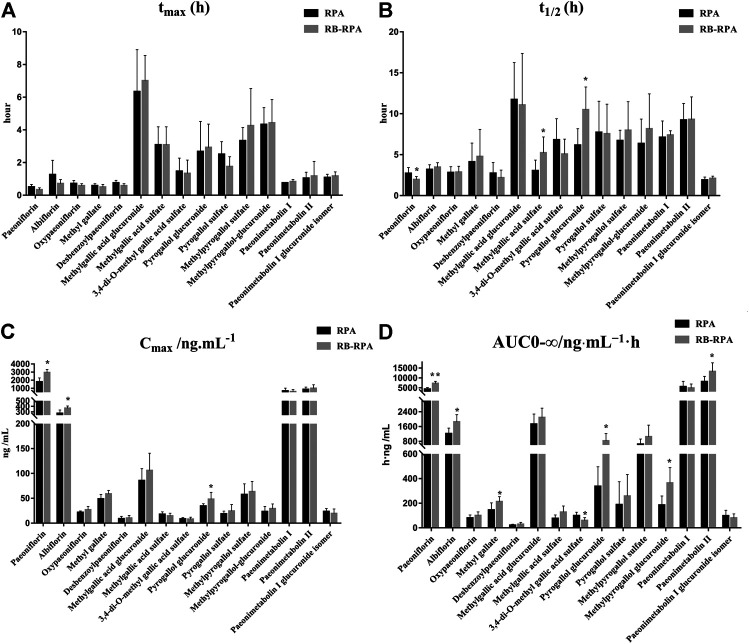
Main pharmacokinetic parameters of 15 compounds from RPA in rat plasma after oral administration of the single extract group (RPA) and the compatibility group (RB-RPA). **(A)** *t*_max_ (h); **(B)** *t*_1/2_ (h); **(C)** *C*_max_ (ng mL^−1^); **(D)** AUC_0-∞_ (ng mL^−1^ h). All data were expressed as mean ± SD, (*n* = 7). *p < 0.05, **p < 0.01 compared with RPA group.

The time–concentration curves of 15 compounds from RPA before and after combination were shown in Figure 4, and the main PK parameters (*C*_max_, *t*_max_, *t*_1/2_, and AUC_0-∞_) were listed in Supplementary Table S7. It was clearly observed that the PK parameters of the combination group were remarkably different from those in RPA group (Figure 5). Specifically, the PK profile of administration of RPA alone was similar to the previous report (Gong et al., 2015). However, compared with the administration of RPA alone, co-administration of RB and RPA was significantly increased the *C*_max_ of paeoniflorin and albiflorin from 1776.42 ± 513.40 to 2932.12 ± 385.77, 271.50 ± 63.29 to 365.94 ± 41.75 ng mL^−1^, respectively (Figure 5C, Supplementary Table S7), which indicated that combination can promote the absorption of paeoniflorin and albiflorin. As a result, the AUC_0-∞_ of paeoniflorin and albiflorin were significantly increased after combination. The results were consistent with previous research that has indicated that saikosaponin A and saikosaponin D could significantly improve the absorption of paeoniflorin and albiflorin in the ileum and colon (Chen et al., 2011), which could be the potential reason to improve the bioavailability of paeoniflorin and albiflorin after combination. As for pyrogallol glucuronide, the *C*_max_, *t*_1/2_, and AUC_0-∞_ of pyrogallol glucuronide were significantly increased to 140.22, 170.29, and 245.38%, respectively, (Figures 5B–D), which indicated that combination of RB and RPA could improve the bioavailability of pyrogallol glucuronide. Besides, the AUC_0-∞_ of methyl gallate, methylpyrogallol glucuronide, and paeonimetabolin II were significantly increased and 3,4-di-*O*-methyl gallic acid sulfate was significantly decreased after combination (Figure 5D). These results indicated that the combination of RB and RPA could significantly improve the bioavailability of six components (paeoniflorin, albiflorin, methyl gallate, pyrogallol glucuronide, methylpyrogallol glucuronide, and paeonimetabolin II) in RPA.

In conclusion, the results demonstrated that the combination of RB and RPA could significantly improve the bioavailability of five components (saikosaponin A, saikosaponin B_2,_ prosaikogenin F, prosaikogenin G, dihydroxyl-dehydrogenation-saikogenin E) in RB, and improve the bioavailability of six components (paeoniflorin, albiflorin, methyl gallate, pyrogallol glucuronide, methylpyrogallol glucuronide and paeonimetabolin II) in RPA. Besides, improving the bioavailability by the combination of RB and RPA could be summarized into two aspects: improvement in the plasma concentration (*C*_max_) and prolongation in system circulation (*t*_1/2_).

### Metabolomics

#### Effect of Radix Bupleuri-Radix Paeoniae Alba Herb Pair Intake on Rat Endogenous Metabolite Endpoints

**FIGURE 6:**
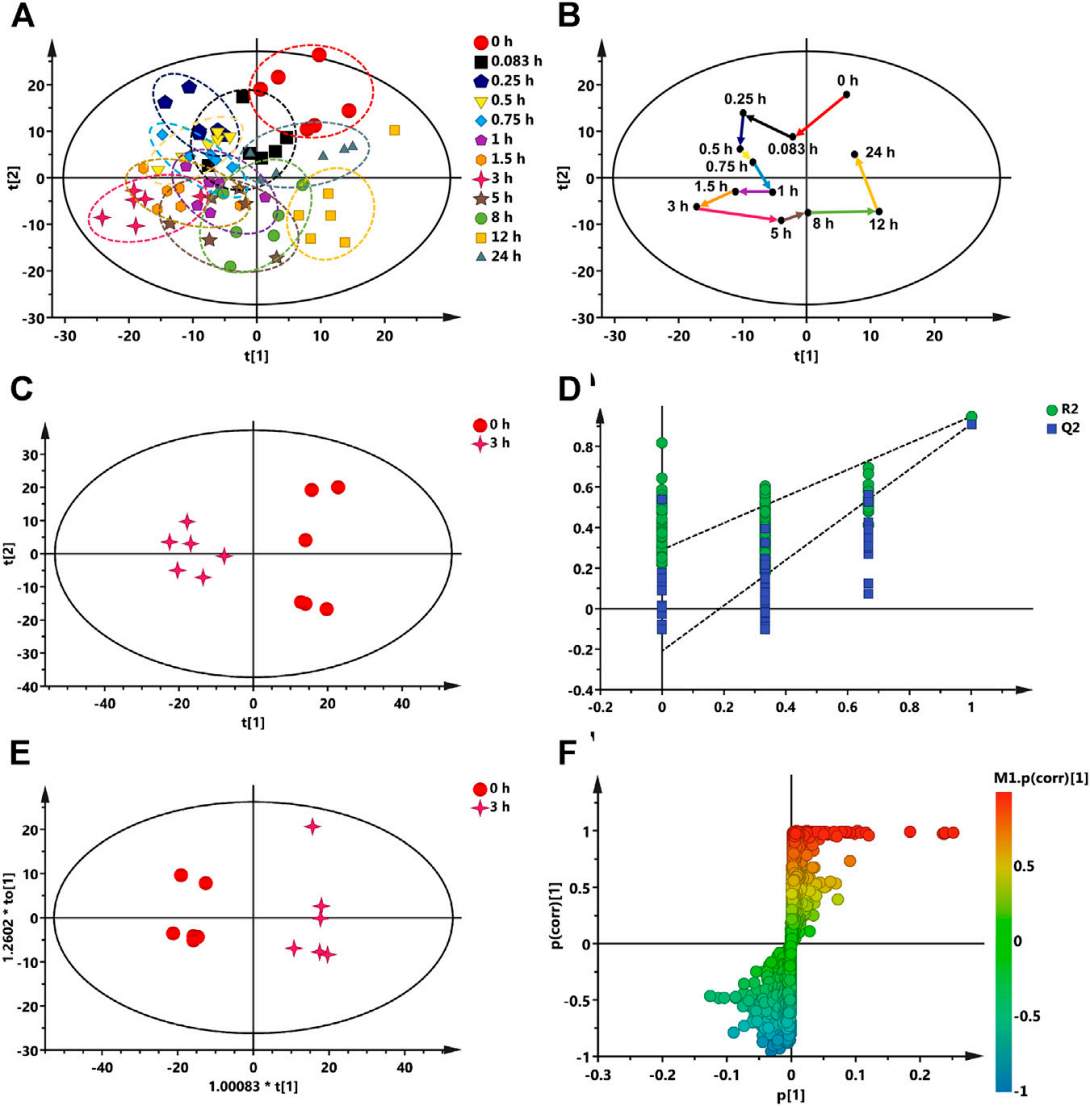
Multivariate data analysis from UPLC-MS/MS. **(A)** The dynamic response profiles of endogenous metabolites intervened by RB-RPA based on PCA score plots. **(B)** A time-dependent trajectory of endogenous metabolite profiles at different time points after RB-RPA intake. **(C)** PCA score plots from 3 h group and 0 h group. **(D)** PLS-DA model validation diagram. **(E)** OPLS-DA score plots from 3 h group and 0 h group. **(F)** S-plot of OPLS-DA.

The plasma samples of rats at different time points after oral administration of RB-RPA were analyzed by UPLC-MS/MS, and the base peak intensity (BPI) chromatograms of plasma samples are shown in Supplementary Figure S3. The metabolomics data acquired from Compound Discoverer 3.0 was imported into SIMCA-P V16.0 for multivariate statistical analysis. The principal component analysis (PCA) was conducted to investigate the trends of endogenous metabolite profiles at different time points after RB-RPA administration. The dynamic response profiles of endogenous metabolites intervened by RB-RPA based on PCA score plots were shown in Figure 6A, and a time-dependent trajectory of endogenous metabolite profiles was shown in Figure 6B. In Figure 6A, each spot represents a plasma sample, and each assembly of samples indicated a specific metabolic profile at different time points. From Figure 6B, endogenous metabolite profiles at different time points after administration were clearly separated from those at the time-point 0 before the RB-RPA intake. The time-dependent trajectory showed that endogenous metabolic profiles underwent a significant change from 0 to 24 h, which may be related to changes in plasma concentrations of RB-RPA components. Furthermore, the endogenous metabolite profiles at 24 h were closed to the pre-dose metabolite profiles, indicating that the metabolic profiles of the subjects showed a recovery trend. Simultaneously, the relative distance calculation between post-dose all time points metabolite profiles and pre-dose metabolite profile from PCA score plot with the average value (x-axis and y-axis) of all samples, to quantify all time points contributions after administration, according to the method described in the literature (Duan et al., 2016), and the results were listed in Supplementary Table S8. As shown in Supplementary Table S8, different relative distance calculation at different time points after administration, indicating that the ability to regulate endogenous metabolic profiles was different, and above all, 3 h after RB-RPA administration displayed the greatest ability to regulate endogenous metabolites as it showed the longest distance calculation. Accordingly, the altered endogenous metabolites associated with RB-RPA were selected by comparing VIP values (VIP >1) and T-test (*p* < 0.05) between the metabolites at time-point 3 h after the RB-RPA intake (3 h group) and the metabolites at time-point 0 h before the RB-RPA intake (0 h group) based on multivariate statistical analysis.

#### Screening and Identification of Endogenous Differential Metabolites Regulated by Radix Bupleuri-Radix Paeoniae Alba

As mentioned above, the endogenous differential metabolites regulated by RB-RPA were selected by comparing the different variables between the 3 h group and the 0 h group based on multivariate statistical analyses. As shown in Figures 6C–F, The PCA score plots indicated that the 3 h group could be obviously separated from the 0 h group (Figure 6C). This finding indicated that 3 h after the RB-RPA intake significantly altered the metabolic fingerprints of plasma compared with the 0 h group. Using the permutation plot test of the PLS-DA model to check the predictive ability and overfitting of the multivariate statistical analysis model (Figure 6D). The permutation test parameters of R^2^X, R^2^Y, and Q^2^ were 0.436, 0.948, and 0.911, respectively. These results showed that the multivariate statistical analysis model had excellent predictive power and had not overfitted. To further enhance the ability of differential metabolite discovery between the 3 h group and 0 h group, the OPLS-DA model was used. The OPLS-DA score plots showed that obvious separation has occurred between the 3 h group and the 0 h group (Figure 6E). The differential metabolites between the 3 h group and the 0 h group were screened by S-plots and VIP values in the OPLS-DA model (Figure 6F), and VIP >1.0 with *p* < 0.05 were considered. Besides, the selected differential metabolites were identified based on the molecular formula, accurate m/z values, MS/MS fragments, and online databases. At last, a total of 21 endogenous differential metabolites were screened and identified (Table 2). Compared with the 0 h, 12 endogenous differential metabolites (DL-ornithine, DL-histidine, choline, gamma-aminobutyric acid, L-glutamic acid, valine, alpha-ketoglutaric acid, L-(-)-asparagine, itaconic acid, picolinic acid, N-acetyl-L-leucine, α-linolenic acid) were significantly increased, and 9 [DL-glutamine, citric acid, L-tyrosine, DL-tryptophan, L-glutathione oxidized, thymidine 5′-monophosphate, taurochenodeoxycholic acid, lysoPC (18:3), cholic acid] were significantly decreased in 3 h group.

**TABLE 2:** Altered endogenous metabolites were detected by UPLC-MS/MS.

No.	Metabolites	T_*R*_ (min)	m/z	Formula	VIP	HMDB ID	3 h/0 h	Ion mode
1	DL-Ornithine	0.706	133.09723	C_5_H_12_N_2_O_2_	1.32	32455	↑*	[M + H]^+^
2	DL-Histidine	0.736	156.07683	C_6_H_9_N_3_O_2_	1.04	00177	↑*	[M + H]^+^
3	Choline	0.820	104.10671	C_5_H_13_NO	1.74	00097	↑**	[M + H]^+^
4	Gamma-Aminobutyric acid	0.856	104.07063	C_4_H_9_NO_2_	1.63	00112	↑**	[M + H]^+^
5	L-Glutamic acid^a^	0.860	146.04568	C_5_H_9_NO_4_	2.13	00148	↑***	[M-H]^−^
6	Valine	0.899	118.08636	C_5_H_11_NO_2_	1.62	00883	↑**	[M + H]^+^
7	DL-Glutamine	0.908	145.06167	C_5_H_10_N_2_O_3_	2.48	00641	↓***	[M-H]^−^
8	Alpha-ketoglutaric acid	0.931	145.01433	C_5_H_6_O_5_	2.32	00208	↑***	[M-H]^−^
9	L-(-)-Asparagine	0.941	115.05036	C_4_H_8_N_2_O_3_	1.03	00168	↑*	[M-H_2_O + H]^+^
10	Citric acid	0.983	191.01963	C_6_H_8_O_7_	2.07	00094	↓***	[M-H]^-^
11	L-Tyrosine^a^	1.032	182.08116	C_9_H_11_NO_3_	2.18	00158	↓***	[M + H]^+^
12	Itaconic acid	1.062	259.04666	C_5_H_6_O_4_	2.52	02092	↑***	[2M-H]^−^
13	Picolinic acid^a^	1.902	124.03942	C_6_H_5_NO_2_	1.96	02243	↑**	[M + H]^+^
14	DL-Tryptophan	3.412	205.09694	C_11_H_12_N_2_O_2_	1.51	13609	↓*	[M + H]^+^
15	N-Acetyl-L-leucine	7.195	172.09740	C_8_H_15_NO_3_	1.72	11756	↑**	[M-H]^−^
16	L-Glutathione oxidized	7.830	613.15913	C_20_H_32_N_6_O_12_S_2_	1.76	03337	↓**	[M + H]^+^
17	Thymidine 5′-monophosphate	11.572	321.04513	C_15_H_16_O_4_P_2_	1.05	01227	↓*	[M-H]^−^
18	Taurochenodeoxycholic acid	12.272	498.28967	C_26_H_45_NO_6_S	1.33	00951	↓*	[M-H]^−^
19	Cholic acid	14.329	407.28021	C_24_H_40_O_5_	2.43	00619	↓***	[M-H]^−^
20	α-Linolenic acid	15.936	279.23164	C_18_H_30_O_2_	1.11	01388	↑*	[M + H]^+^
21	LysoPC (18:3)	17.149	518.32404	C_26_H_48_NO_7_P	1.04	10387	↓*	[M + H]^+^

“↓” or “↑” means the metabolite significantly decreased or increased in 3 h group compared with 0 h group.

*p < 0.05, **p < 0.01, ***p < 0.001 compared with 0 h group.

^a^ Validated with standard.

**FIGURE 7:**
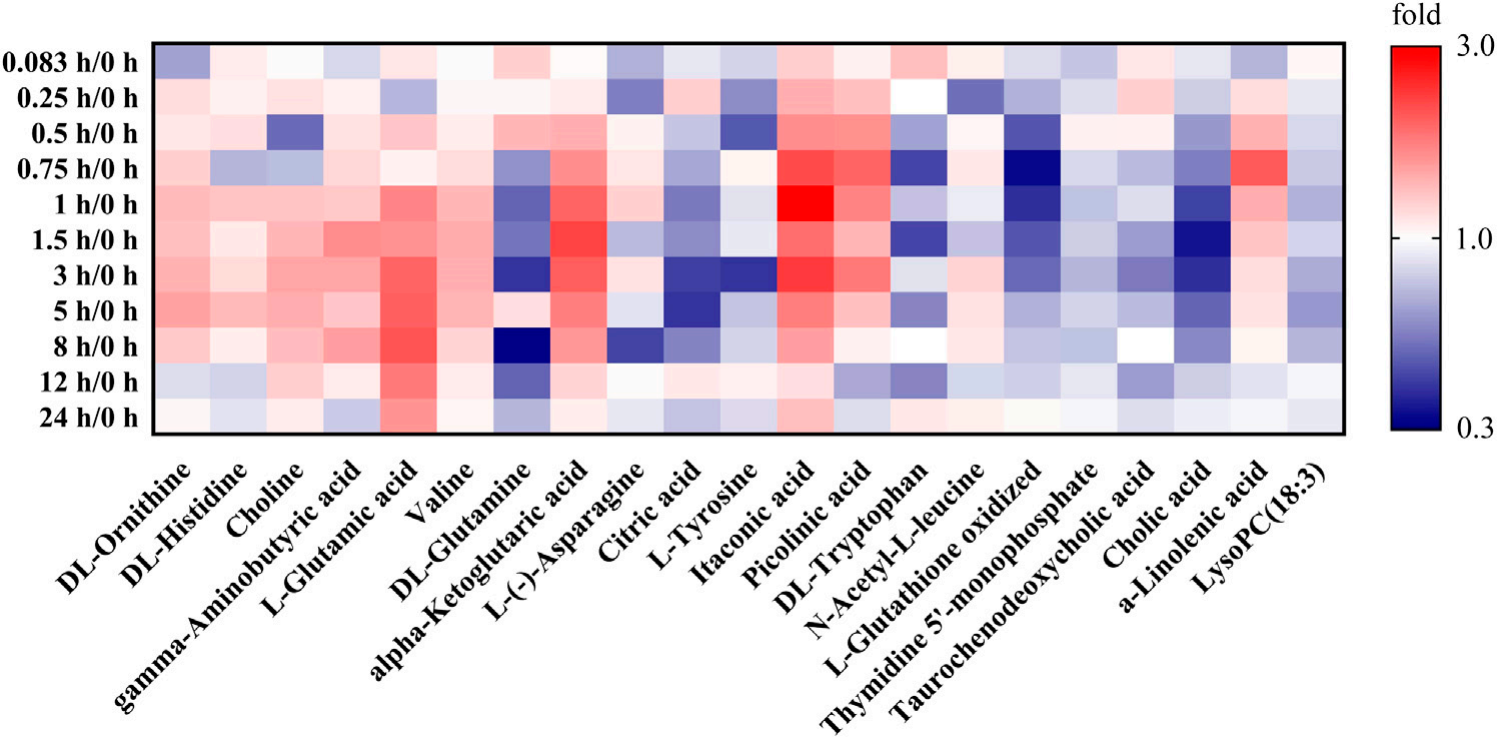
Effect of RB-RPA herb pair intake on rat metabolite endpoints. Each cell in the heat map represents the fold change between at each time points after administration and at time-point 0 before the RB-RPA intake for a particular metabolite. The red color indicates that the ratio is greater than 1, and the blue color indicates that the ratio is less than 1. It visualizes the level of each metabolite at each time points ranging from high (red) over average (white) to low (blue).

Based on the investigation of metabonomics and the semi-quantification, the relative peak areas of endogenous differential metabolites were calculated at various time points before and after administration. The detailed data was listed in Supplementary Table S9. Furthermore, Metabonomic response profiles response to RB-RPA intervention at various time points after administration were depicted as a heat map (Figure 7). In heat map, each cell represents the fold change between the two time points for a particular endogenous differential metabolite. These results indicated that 21 endogenous differential metabolites exhibited varying degrees of dynamic changes after RB-RPA administration.

### The Potential Link Between Radix Bupleuri-Radix Paeoniae Alba Phytochemicals and the Altered Endogenous Metabolites

**FIGURE 8:**
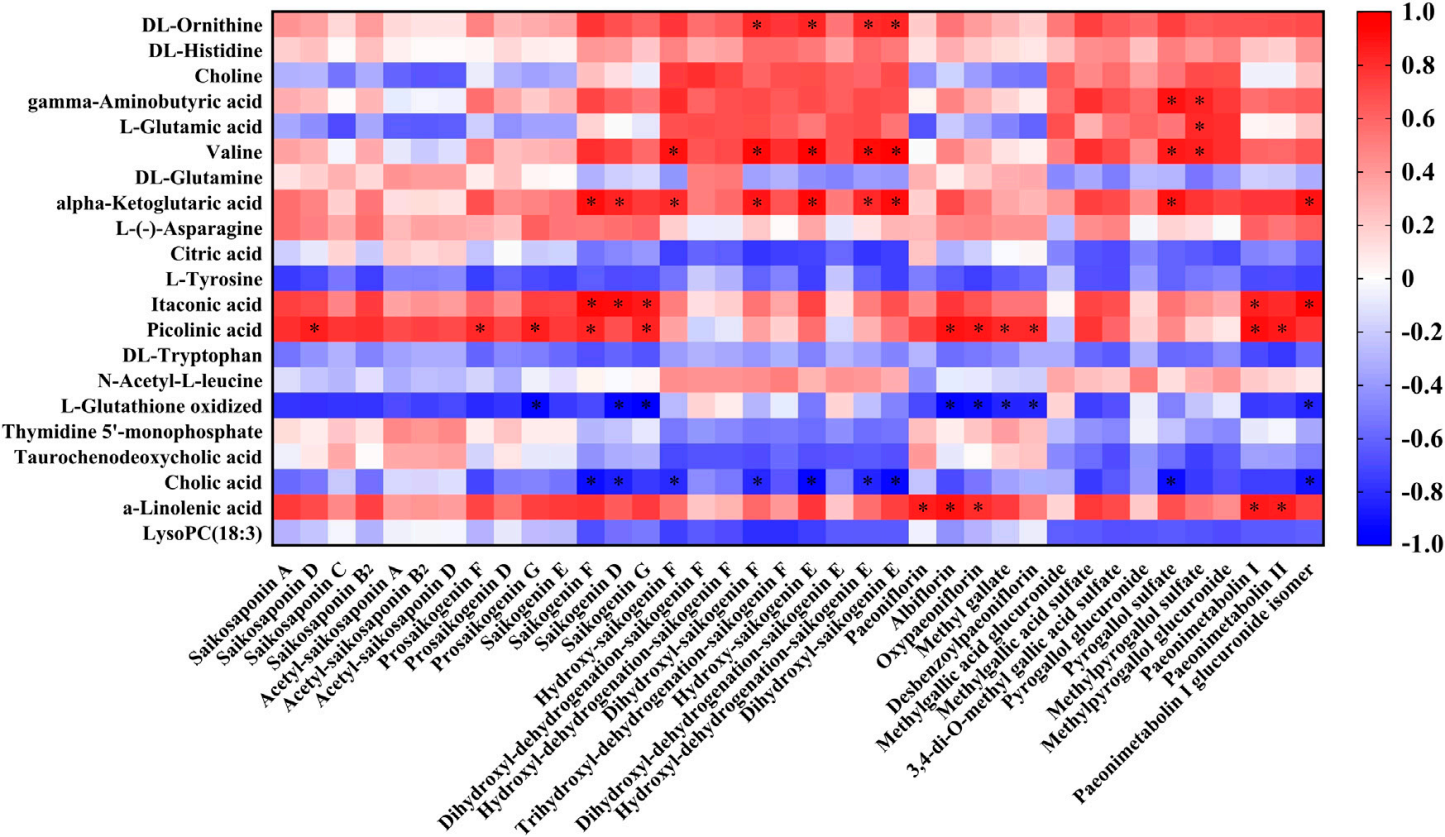
Correlation analysis between the 38 RB-RPA herb pair phytochemicals and the altered 21 endogenous metabolites according to Pearson correlation coefficient. Red color indicated that |r| was a positive value and blue indicated that |r| was a negative value. The darker the color, the larger the |r| value. * represents *p* < 0.05 and |r| >0.8.

The correlation between the RB-RPA concentration-time curves of 38 components and the dynamic response profile of altered 21 endogenous metabolites was presented in Figure 8, with positive (red color) and negative (blue color) (*p* < 0.05, |r| >0.8) values. Correlation analysis demonstrated that RB-RPA herb pair phytochemicals had an impact on endogenous metabolites. In general, the change of the plasma concentration of endogenous metabolites in response to the alteration of the bioavailability of RB-RPA phytochemicals. As shown in Figure 8, most of RB-RPA phytochemicals were positively correlated with DL-ornithine, gamma-aminobutyric acid, valine, alpha-ketoglutaric acid, itaconic acid, picolinic acid, α-linolenic acid, and were negatively correlated with L-glutathione oxidized and cholic acid.

**FIGURE 9:**
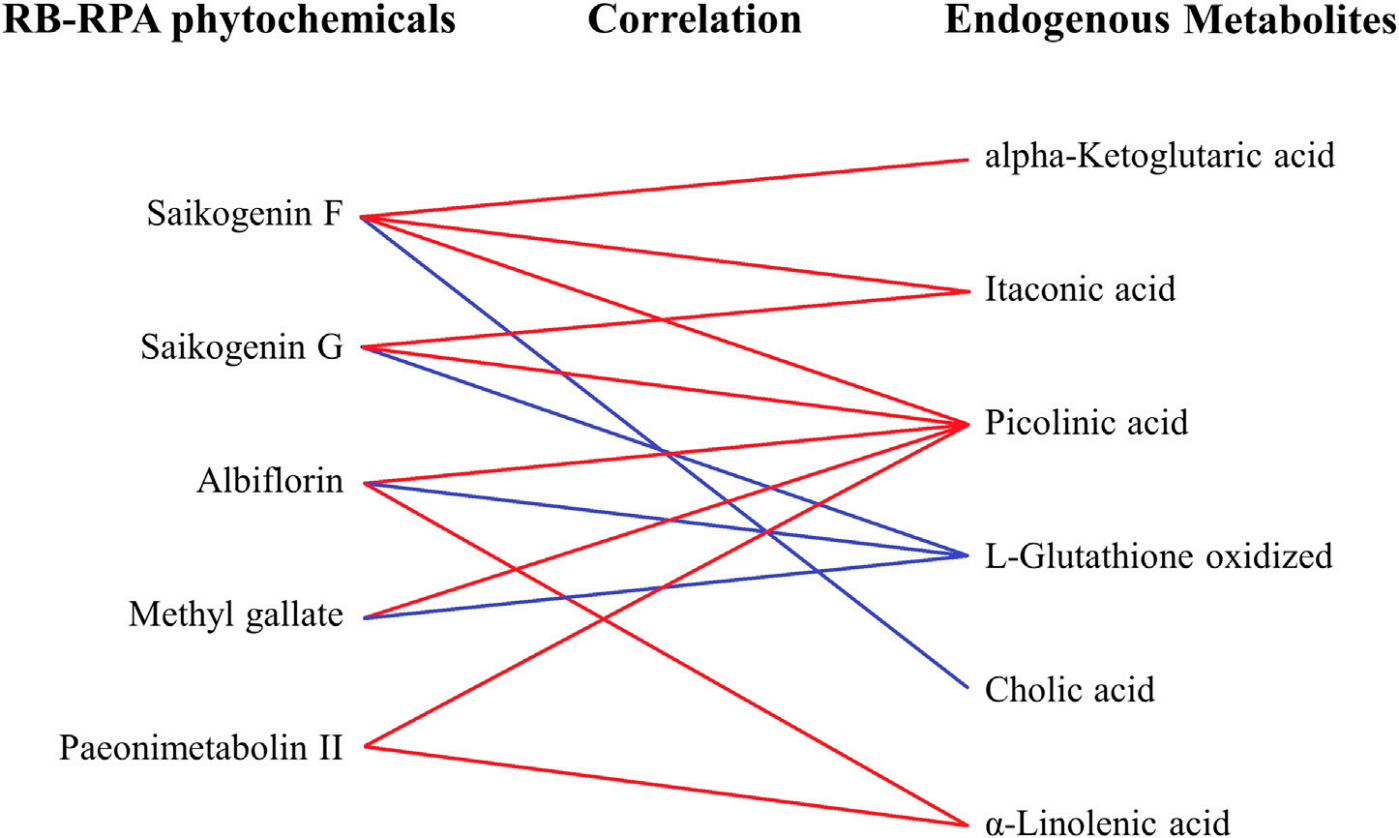
The correlation between the phytochemicals improved bioavailability after compatibility and the altered endogenous metabolites. The relationships among the phytochemicals and endogenous metabolites were visualized in the form of correlation maps, which are displayed by red (positive) or blue (negative) lines.

Besides, to investigate the effects of the combination of RB and RPA on endogenous metabolites, the correlation between the phytochemicals improved bioavailability after combination and endogenous metabolites was summarized. As shown in Figure 9, the components for bioavailability significantly improved after combination such as saikogenin F, saikogenin G, albiflorin, methyl gallate, paeonimetabolin II were significantly positively correlated with picolinic acid; saikogenin F, saikogenin G were significantly positively correlated with itaconic acid; albiflorin, paeonimetabolin II were significantly positively correlated with α-linolenic acid; saikogenin G, albiflorin, methyl gallate were significantly negatively correlated with L-glutathione oxidized; saikogenin F was significantly positively correlated with alpha-ketoglutaric acid and was significantly negatively correlated with cholic acid. It was worth noting that picolinic acid and α-linolenic acid were endogenous metabolites with a strong neuroprotective effect, and itaconic acid was endogenous metabolites with anti-inflammatory activity. These results indicated that the combination of RB and RPA can enhance each other’s neuroprotective and anti-inflammatory activities.

#### Picolinic Acid

Picolinic acid is an end-product of the kynurenine pathway with a strong neuroprotective effect (Lovelace et al., 2017). Recent research has indicated that picolinic acid showed antidepressant effects by decreasing the immobile time of forced swim test and reversing the significant rise in plasma corticosterone level in CUMS-induced depression rats (Dubey et al., 2015). Furthermore, as well as clinically, decreased plasma picolinic acid levels have been demonstrated in depressed patients (Ryan et al., 2020), and picolinic acid has also been shown to produce significant antidepressant effects in a typical depression (Davidson et al., 2003). Our study demonstrated that the five phytochemicals with improved bioavailability after combination (saikogenin F, saikogenin G, albiflorin, methyl gallate, paeonimetabolin II) were significantly positively correlated with picolinic acid, a metabolite that was significantly downregulated in depressed patients (Colle et al., 2020). Besides, chronic stress can cause imbalances in the kynurenine metabolic pathway and excessively produce the neurotoxic product quinolinic acid, thereby promoting the occurrence of depressive behavior (Won and Kim., 2016). It was worth noting that as a neuroprotective product, picolinic acid has also been shown to antagonize the adverse effects of quinolinic acid on the nervous system to prevent depression and anxiety symptoms (Grant et al., 2009). These results indicated that these five phytochemicals can inhibit the neurotoxic effects of quinolinic acid and maintain the balance of the kynurenine metabolic pathway by acting with picolinic acid, thereby avoiding the occurrence of depressive behavior. Besides, several studies have shown that RB, RPA, and their main active ingredients (such as saikosaponins, albiflorin, methyl gallate, etc.) have significant neuroprotective and antidepressant activity (Wang et al., 2016; Li et al., 2017; Li et al., 2018). Therefore, we speculated that the combination of RB and RPA by increasing the bioavailability of these five phytochemicals, the impact on picolinic acid was strengthened to enhance each other’s neuroprotective.

#### Itaconic Acid

Itaconic acid is a crucial anti-inflammatory endogenous metabolite, which was produced by the decarboxylation of *cis*-aconitate, a tricarboxylic acid cycle intermediate (Michelucci et al., 2013), previous studies have found that itaconate exerts anti-inflammatory effects by inhibiting succinate dehydrogenase (Bordon, 2018). Additionally, recent studies have confirmed that itaconic acid was required for the activation of the anti-inflammatory transcription factor Nrf2 by lipopolysaccharide in macrophages, enabling Nrf2 to increase the expression of downstream genes with anti-oxidant and anti-inflammatory capacities (Mills et al., 2018). Further, RB has also been proven to exert anti-inflammatory effects by regulating the Nrf2 signaling pathway (Jia et al., 2019). Our study suggests that the two phytochemicals with improved bioavailability after RB and RPA combination (saikogenin F, saikogenin G) were significantly positively correlated with itaconic acid. The saikogenin F, saikogenin G were deglycosylated metabolites of saikosaponin A and saikosaponin D transformed by intestinal bacteria in the gastrointestinal tract (Shimizu et al., 1985). In addition, related studies speculated that saikosaponins, the main component of RB, was transformed into saikogenins by human intestinal flora, and then exerts pharmacological activity (Liu et al., 2019). These research results indicated that compared to saikosaponin A and saikosaponin D, the pharmacological activity of saikogenin F and saikogenin G in the body may be more significant. And the saikogenin F and saikogenin G maybe activate the Nrf2 signaling pathway by acting on itaconic acid, thereby exerting an anti-inflammatory effect. Meanwhile, saikogenins have also been reported to have anti-inflammatory pharmacological activity (Cheng and Tsai, 1986; Toriniwa et al., 2006), which is in accordance with our research. we speculated that the combination of RB and RRA can enhance the anti-inflammatory effect of saikogenin F and saikogenin G.

#### α-linolenic Acid

The α-linolenic acid is a polyunsaturated omega-3 fatty acid whose metabolism in the body has been well characterized. When α-linolenic acid was ingested, the body converts it to long-chain polyunsaturated fatty acids: eicosapentaenoic acid and docosahexaenoic acid, both of which were considered to exert strong actions on brain functions (Connor, 1999). BDNF is a neurotrophin, it’s known for its effects on promoting neurogenesis and neuronal survival, which is significantly associated with depression (Oh et al., 2019). Studies have found that oral consumption of α-linolenic acid increases serum BDNF levels in healthy adults, which may be due to the neuroprotective impact of eicosapentaenoic acid and docosahexaenoic acid on the nervous system (Hadjighassem et al., 2015). Besides, chronic dietary α-linolenic acid deficiency alters dopaminergic and serotoninergic neurotransmission (Delion et al., 1994), which finally accelerates the development of depression. The albiflorin and paeonimetabolin II were the prototype components and metabolites in RPA, respectively. Our study suggests that the two phytochemicals with improved bioavailability after RB and RPA combination (albiflorin, paeonimetabolin II) were significantly positively correlated with α-linolenic acid. It has been reported that albiflorin, the main active component of RPA, can be used as inhibitors of D-amino acid oxidase in the brain, improved brain function and exerted antidepressant activity (Zhao et al., 2018). In addition, this experiment also found that the combination of RB and RPA can enhance the regulation of α-linolenic acid to enhance the antidepressant activity of RPA. However, it was not clear how RPA and albiflorin can improve brain function and exert antidepressant activity by regulating α-linolenic acid.

## Conclusion

In conclusion, RB and RPA compatibility could significantly improve the bioavailability of five components in RB, and improve the bioavailability of six components in RPA, which could be summarized into two aspects: improvement in the plasma concentration (*C*_max_) and prolongation in system circulation (*t*_1/2_). Furthermore, “plant metabolome” and “body metabolome” correlation analysis results indicated that compatibility of RB and RPA can enhance each other’s neuroprotective and anti-inflammatory activities, which provided a research basis for further research on the synergistic pharmacological mechanism of the compatibility of RB and RPA.

## Data Availability

The original contributions presented in the study are included in the article/Supplementary Material, further inquiries can be directed to the corresponding author.
