# Supplementary material for: Studies on the Changes of Pharmacokinetics Behaviors of Phytochemicals and the Influence on Endogenous Metabolites After the Combination of Radix Bupleuri and Radix Paeoniae Alba Based on Multi-Component Pharmacokinetics and Metabolomics
Source: Front Pharmacol. 2021 Mar 8;12:630970. doi: 10.3389/fphar.2021.630970 (PMC7982521; doi:10.3389/fphar.2021.630970)
Supplement: Supplementary file 1 [file datasheet1.docx]

Supplementary Material

**Studies on the changes of pharmacokinetics behaviors of phytochemicals and the influence on endogenous metabolites after the combination of Radix Bupleuri and Radix Paeoniae Alba based on multi-component pharmacokinetics and metabolomics**

Congcong Chen ^1, 2^, Qicai Yin ^1, 2^, Junshen Tian ^1^, Xiaoxia Gao ^1^,

Xuemei Qin ^1^, Guanhua Du ^1,^ ^3^, Yuzhi Zhou ^1,*^

^1^ *Modern Research Center for Traditional Chinese Medicine of Shanxi University, No.92, Wucheng Road, Taiyuan* *030006, People’s Republic of China;*

^2^ *College of Chemistry and Chemical Engineering, Shanxi University, No. 92,*

*Wucheng Road, Taiyuan 030006, P. R. China*

^3^ *Institute of Materia Medica, Chinese Academy of Medical Sciences & Peking Union Medical College, Beijing 100050,* *P. R. China*

**^*^** **Correspondence authors.** Tel: +86-351-7011202; Fax: +86-351-7011202

E-mail addresses: [zhouyuzhi@sxu.edu.cn](mailto:zhouyuzhi@sxu.edu.cn) (Yu-Zhi Zhou);

**Supplementary Files:**

**Fig. S1**. The base peak chromatograms of herb extracts and mixed standards (saikosaponin A: SSa, saikosaponin D: SSd, saikosaponin C: SSc, saikosaponin B_2_: SSb_2_, paeoniflorin, albiflorin, oxypaeoniflorin, methyl gallate, and the Internal standard). A: Radix Bupleuri extract; B: Radix Paeoniae Alba extract; C: mixture of Radix Bupleuri and Radix Paeoniae Alba extract; D: mixed standards.

**Table S1**. The content of eight compounds in single extract and mixture extract. Radix Bupleuri extract: RB; Radix Paeoniae Alba extract: RPA; mixture of Radix Bupleuri and Radix Paeoniae Alba extract: RB-RPA. All data were expressed as mean ± SD, (*n* = 6).

**Fig. S2**. Extracted-ion [chromatograms](https://www.sciencedirect.com/topics/medicine-and-dentistry/chromatography) of SSa, SSd, and SSb_2_ (I), SSc (II), paeoniflorin and albiflorin (III), oxypaeoniflorin (IV), methyl gallate (V). (A) Blank plasma; (B) drug-containing plasma after administration of Radix Bupleuri extract; (C) drug-containing plasma after administration of Radix Paeoniae Alba extract; (D) drug-containing plasma after administration of mixture of Radix Bupleuri and Radix Paeoniae Alba extract; (E) blank plasma spiked with reference compound.

**Table S2**. Liner range, regression equation, correlation coefficient for eight compounds.

**Table S3**. Recovery and the matrix effect of eight target analytes in QC plasma samples (*n* = 6).

**Table S4**. Precision and accuracy of eight target analytes in QC plasma samples (mean ± SD, *n* = 6).

**Table S5**. Stability of eight target analytes in QC plasma samples (mean ± SD, *n* = 5).

**Table S6**. Main pharmacokinetic parameters of the 23 compounds from RB in rat plasma after oral administration of the single extract (RB) and the mixed extracts (RB-RPA). All data were expressed as mean ± SD, (*n* = 7).

**Table S7**. Main pharmacokinetic parameters of the 15 compounds from RPA in rat plasma after oral administration of the single extract (RPA) and the mixed extracts (RB-RPA). All data were expressed as mean ± SD, (*n* = 7).

**Fig. S3**. UPLC-MS/MS base peak chromatograms of plasma samples after oral administration of RB-RPA

**Table S8**. Relative distance calculation between post-dose all time points metabolite profiles and pre-dose metabolite profile from the score plot of PCA with average value (x-axis and y-axis) of all samples. Values are presented as mean± SD, (*n* = 6).

**Table S9**. List of the altered endogenous metabolites in response to RB-RPA intervention and the data were normalized by IS (glycyrrhizin). Values are presented as mean± SD, (*n* = 6).


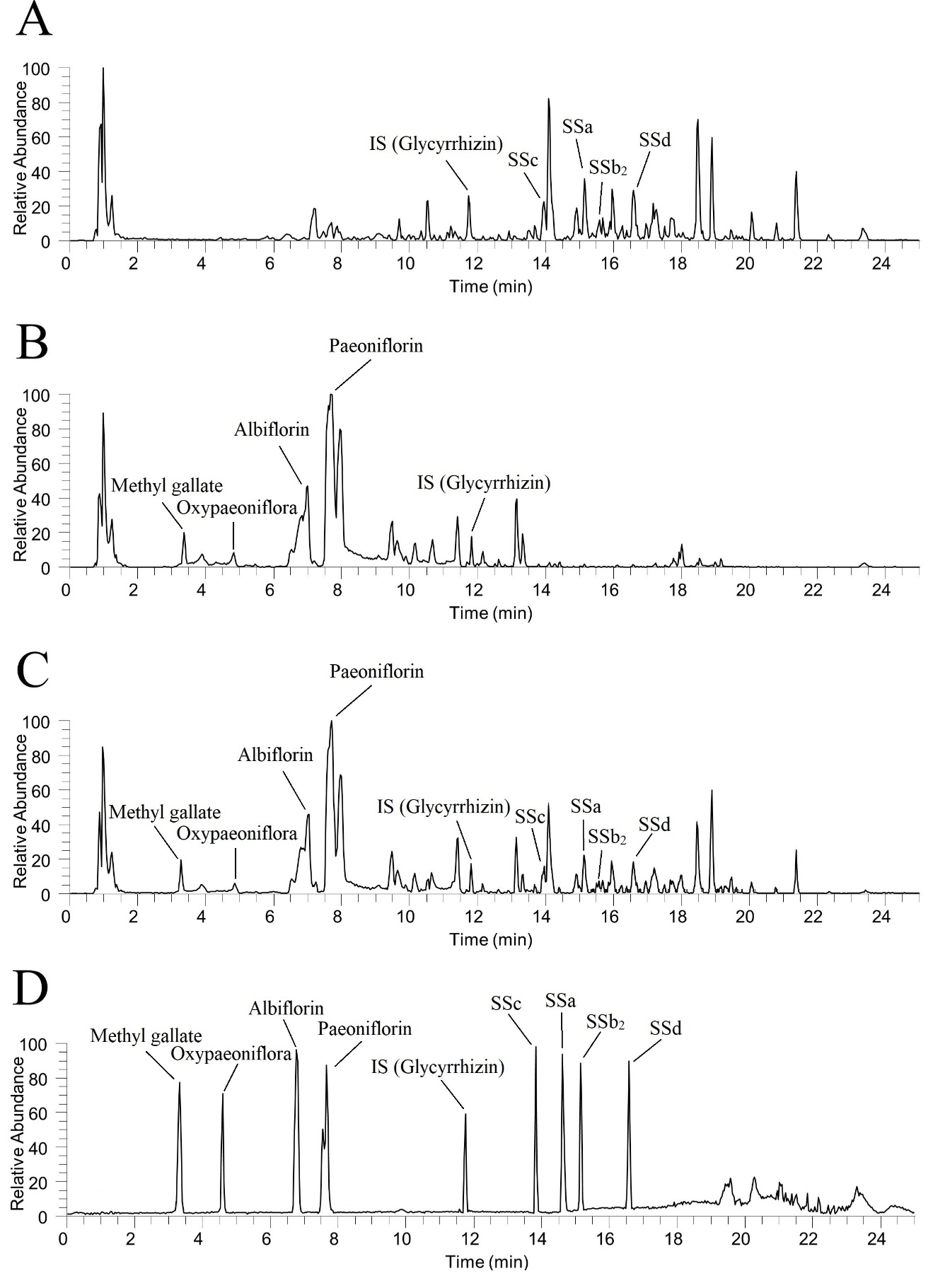


**Fig. S1**. The base peak chromatograms of herb extracts and mixed standards (saikosaponin A: SSa, saikosaponin D: SSd, saikosaponin C: SSc, saikosaponin B_2_: SSb_2_, paeoniflorin, albiflorin, oxypaeoniflorin, methyl gallate, and the Internal standard). A: Radix Bupleuri extract; B: Radix Paeoniae Alba extract; C: mixture of Radix Bupleuri and Radix Paeoniae Alba extract; D: mixed standards.

**Table S1**. The content of eight compounds in single extract and mixture extract. Radix Bupleuri extract: RB; Radix Paeoniae Alba extract: RPA; mixture of Radix Bupleuri and Radix Paeoniae Alba extract: RB-RPA. All data were expressed as mean ± SD, (*n* = 6).

| Compounds | RB (mg/ml) | RPA (mg/ml) | RB-RPA (mg/ml) |
| --- | --- | --- | --- |
| Saikosaponin A | 5.195±0.008 | 0 | 5.181±0.011 |
| Saikosaponin D | 3.925±0.006 | 0 | 3.911±0.007 |
| Saikosaponin C | 2.091±0.004 | 0 | 2.082±0.007 |
| Saikosaponin B_2_ | 1.082±0.003 | 0 | 1.066±0.006 |
| Paeoniflorin | 0 | 38.751±0.037 | 38.982±0.041 |
| Albiflorin | 0 | 6.463±0.010 | 6.531±0.008 |
| Oxypaeoniflorin | 0 | 0.673±0.005 | 0.691±0.005 |
| Methyl gallate | 0 | 1.613±0.008 | 1.625±0.007 |


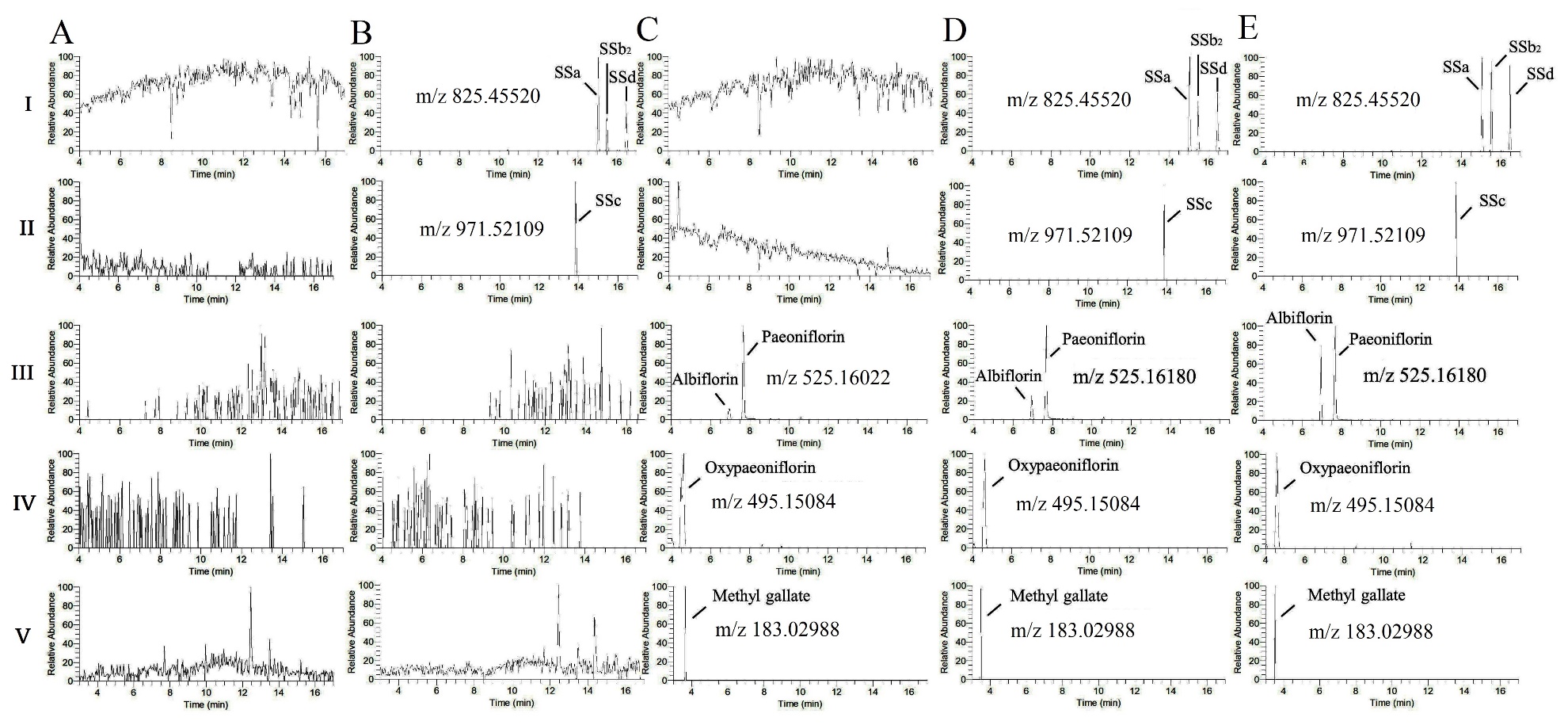


**Fig. S2**. Extracted-ion [chromatograms](https://www.sciencedirect.com/topics/medicine-and-dentistry/chromatography) of SSa, SSd, and SSb_2_ (I), SSc (II), paeoniflorin and albiflorin (III), oxypaeoniflorin (IV), methyl gallate (V). (A) Blank plasma; (B) drug-containing plasma after administration of Radix Bupleuri extract; (C) drug-containing plasma after administration of Radix Paeoniae Alba extract; (D) drug-containing plasma after administration of mixture of Radix Bupleuri and Radix Paeoniae Alba extract; (E) blank plasma spiked with reference compound.

**Table S2**. Liner range, regression equation, correlation coefficient for eight compounds.

| Compounds | Liner range (ng/ml) | Regression equation | Correlation coefficient (*r^2^*) | LLOQ ng/ml |
| --- | --- | --- | --- | --- |
| Saikosaponin A | 0.2-500 | *y* = 0.0541*x* + 0.0246 | 0.9993 | 0.2 |
| Saikosaponin D | 0.1-250 | *y* = 0.0231*x* + 0.0311 | 0.9968 | 0.1 |
| Saikosaponin C | 0.1-250 | *y* = 0.0384*x* + 0.0421 | 0.9982 | 0.1 |
| Saikosaponin B_2_ | 0.1-250 | *y* = 0.0263*x* + 0.0289 | 0.9985 | 0.1 |
| Paeoniflorin  Albiflorin  Oxypaeoniflorin  Methyl gallate | 2-5000  0.5-1250  0.1-250  0.2-500 | *y* = 0.0943*x* + 0.0747  *y* = 0.1484*x* + 0.0517  *y* = 0.1013*x* + 0.0933  *y* = 0.0762*x* + 0.0046 | 0.9996  0.9994  0.9979  0.9991 | 2  0.5  0.1  0.2 |

**Table S3**. Recovery and the matrix effect of eight target analytes in QC plasma samples (*n* = 6).

| Compounds | QC conc.  (ng/ml) | Recovery (%, mean ± SD) | Matrix effect (%, mean ± SD) |
| --- | --- | --- | --- |
| Saikosaponin A | 100 | 98.84±2.12 | 96.41±1.97 |
|  | 10 | 94.13±2.48 | 97.75±2.28 |
|  | 0.4 | 90.96±3.39 | 92.82±2.86 |
| Saikosaponin D | 50 | 104.63±3.19 | 93.14±3.62 |
|  | 5 | 95.47±3.81 | 95.23±1.92 |
|  | 0.2 | 89.88±3.56 | 91.74±2.81 |
| Saikosaponin C | 50 | 86.27±5.14 | 104.92±4.19 |
|  | 5 | 87.42±3.68 | 91.59±4.83 |
|  | 0.2 | 85.83±2.95 | 87.75±3.22 |
| Saikosaponin B_2_ | 50 | 93.81±1.77 | 104.45±2.33 |
|  | 5 | 86.75±3.41 | 103.85±2.18 |
|  | 0.2 | 81.32±5.59 | 82.8±5.96 |
| Albiflorin | 250 | 105.32±3.75 | 93.73±2.34 |
|  | 20 | 89.23±4.37 | 84.38±4.50 |
|  | 1 | 91.86±5.23 | 94.04±3.96 |
| Paeoniflorin | 1000 | 98.59±2.25 | 99.33±1.42 |
|  | 100 | 97.32±1.96 | 96.38±1.84 |
|  | 4 | 98.93±1.58 | 98.75±2.13 |
| Oxypaeoniflorin | 50 | 103.39±3.77 | 82.383±5.33 |
|  | 5 | 105.22±3.25 | 79.61±7.22 |
|  | 0.2 | 106.83±6.27 | 85.53±5.85 |
| Methyl gallate | 100 | 102.51±2.27 | 98.2±2.83 |
|  | 10 | 95.34±3.94 | 106.26±3.88 |
|  | 0.4 | 97.23±2.45 | 111.53±4.32 |

**Table S4.** Precision and accuracy of eight target analytes in QC plasma samples (mean ± SD, *n* = 6).

| Compounds | QC conc.  (ng/ml) | Intra-day | | | Inter-day | | |
| --- | --- | --- | --- | --- | --- | --- | --- |
|  |  | Calc. conc. (ng/ml) | Precision  RSD (%) | Accuracy  RE (%) | Calc. conc. (ng/ml) | Precision  RSD (%) | Accuracy  RE (%) |
| Saikosaponin A | 100 | 96.96±6.39 | 6.59 | -3.04 | 95.68±7.96 | 8.32 | -4.32 |
|  | 10 | 9.55±0.76 | 7.96 | -4.49 | 10.81±0.63 | 5.83 | 8.12 |
|  | 0.4 | 0.41±0.03 | 7.18 | 2.42 | 0.42±0.04 | 9.09 | 5.23 |
| Saikosaponin D | 50 | 52.12±3.11 | 5.97 | 4.24 | 52.92±4.38 | 8.28 | 5.84 |
|  | 5 | 4.82±0.28 | 5.82 | 3.58 | 4.93±0.13 | 2.64 | -1.4 |
|  | 0.2 | 0.21±0.02 | 9.11 | 8.12 | 0.22±0.02 | 9.81 | 8.02 |
| Saikosaponin C | 50 | 48.23±1.43 | 2.96 | -3.54 | 48.02±2.48 | 5.16 | -3.96 |
|  | 5 | 4.77±0.22 | 4.61 | -4.58 | 4.65±0.35 | 7.53 | -7.11 |
|  | 0.2 | 0.18±0.02 | 9.06 | -8.05 | 0.18±0.02 | 10.04 | -8.11 |
| Saikosaponin B_2_ | 50 | 49.62±1.13 | 2.27 | -0.76 | 48.12±3.58 | 7.44 | -3.76 |
|  | 5 | 4.75±0.11 | 2.32 | -4.98 | 4.81±0.12 | 2.49 | --3.84 |
|  | 0.2 | 0.19±0.01 | 8.34 | -7.98 | 0.21±0.02 | 11.48 | 7.93 |
| Albiflorin | 250 | 252.52±9.29 | 3.68 | 1.01 | 249.31±11.52 | 4.62 | -0.28 |
|  | 25 | 24.94±1.26 | 4.35 | -0.24 | 24.12±1.56 | 6.46 | -3.52 |
|  | 1 | 1.06±0.08 | 7.55 | 6.01 | 1.04±0.07 | 6.73 | 4.34 |
| Paeoniflorin | 1000 | 986.28±54.25 | 5.50 | -1.37 | 991.23±42.94 | 4.33 | 0.88 |
|  | 100 | 103.34±1.88 | 1.82 | 3.34 | 97.21±2.14 | 2.21 | -2.79 |
|  | 4 | 4.21±0.33 | 7.84 | 5.25 | 3.78±0.29 | 7.67 | -5.48 |
| Oxypaeoniflorin | 50 | 50.65±1.21 | 2.39 | 1.3 | 51.43±3.53 | 6.86 | 2.86 |
|  | 5 | 5.24±4.23 | 8.07 | 4.8 | 5.21±0.32 | 6.14 | 4.22 |
|  | 0.2 | 0.19±0.01 | 8.95 | -7.78 | 0.20±0.01 | 9.23 | 3.24 |
| Methyl gallate | 100 | 104.82±2.42 | 2.31 | 4.82 | 105.32±3.15 | 2.99 | 5.32 |
|  | 10 | 9.48±0.52 | 5.49 | -5.19 | 9.18±0.83 | 9.04 | -0.82 |
|  | 0.4 | 0.41±0.04 | 9.09 | 2.81 | 0.42±0.05 | 11.42 | 5.89 |

**Table S5.** Stability of eight target analytes in QC plasma samples (mean ± SD, *n* = 5).

| Compounds | QC conc.  (ng/ml) | Long-term stability  -20°C for 30 d | | Freeze-thaw stability  3 freeze-thaw cycles | | Short-term stability  24 h in room temperature | | Post-preparative stability  24 h in the autosampler (4°C) | |
| --- | --- | --- | --- | --- | --- | --- | --- | --- | --- |
|  |  | Calc. conc. (ng/ml) | Accuracy  RE (%) | Calc. conc. (ng/ml) | Accuracy  RE (%) | Calc. conc. (ng/ml) | Accuracy  RE (%) | Calc. conc. (ng/ml) | Accuracy  RE (%) |
| Saikosaponin A | 100 | 94.67±5.36 | -5.33 | 93.84±7.25 | -6.56 | 92.89±7.86 | -7.11 | 95.81±3.53 | -4.19 |
|  | 10 | 9.24±0.63 | -7.61 | 10.19±0.88 | 1.92 | 9.11±0.78 | -8.91 | 9.42±0.24 | -5.85 |
|  | 0.4 | 0.38±0.03 | -4.81 | 0.38±0.03 | -5.53 | 0.36±0.03 | -8.72 | 0.41±0.03 | 2.51 |
| Saikosaponin D | 50 | 52.78±4.23 | 5.56 | 52.98±4.14 | 5.97 | 46.35±4.32 | -7.33 | 51.04±2.83 | 2.08 |
|  | 5 | 4.83±0.36 | -3.38 | 5.18±0.43 | 3.63 | 4.71±0.34 | -5.82 | 4.92±0.32 | -1.63 |
|  | 0.2 | 0.19±0.01 | -7.98 | 0.19±0.01 | -5.26 | 0.18±0.01 | -8.73 | 0.19±0.01 | -6.43 |
| Saikosaponin C | 50 | 48.12±2.25 | -3.76 | 52.02±3.54 | 4.04 | 47.52±3.24 | -4.96 | 48.83±2.08 | -2.34 |
|  | 5 | 4.92±0.21 | -1.61 | 4.83±0.33 | -3.41 | 4.77±0.27 | -4.64 | 5.11±0.36 | 2.26 |
|  | 0.2 | 0.19±0.01 | -0.82 | 0.21±0.02 | 5.11 | 0.19±0.01 | -8.64 | 0.19±0.01 | -8.17 |
| Saikosaponin B_2_ | 50 | 49.31±3.22 | -1.38 | 48.97±4.02 | -2.06 | 48.02±3.76 | -3.96 | 50.83±1.43 | 1.66 |
|  | 5 | 4.89±0.18 | -2.27 | 4.84±0.22 | -3.23 | 4.72±0.24 | -5.62 | 4.81±0.22 | -3.82 |
|  | 0.2 | 0.19±0.01 | -7.87 | 0.19±0.01 | -8.21 | 0.19±0.01 | -8.32 | 0.20±0.00 | -3.18 |
| Albiflorin | 250 | 253.35±15.55 | 1.34 | 252.24±16.45 | -0.90 | 253.42±15.43 | 1.37 | 256.64±17.53 | 2.66 |
|  | 25 | 26.35±1.88 | 5.41 | 26.57±2.65 | 6.28 | 26.53±3.23 | 6.12 | 26.04±3.13 | 4.16 |
|  | 1 | 1.04±0.08 | 4.51 | 0.96±0.06 | -4.13 | 0.91±0.04 | -8.92 | 0.93±0.05 | -6.55 |
| Paeoniflorin | 1000 | 988.53±53.63 | -1.15 | 979.24±58.46 | -2.08 | 971.37±60.52 | -2.86 | 991.03±42.65 | 0.99 |
|  | 100 | 106.35±8.14 | 6.35 | 94.53±8.03 | -5.47 | 107.35±9.34 | 7.35 | 96.35±6.34 | -3.65 |
|  | 4 | 3.89±0.17 | -2.75 | 3.77±0.22 | -5.75 | 3.71±0.24 | -7.25 | 3.91±0.11 | -2.25 |
| Oxypaeoniflorin | 50 | 49.24±2.95 | -1.52 | 48.78±3.76 | -2.44 | 46.32±4.11 | -7.36 | 48.52±3.48 | -2.96 |
|  | 5 | 5.11±0.29 | 2.24 | 4.87±0.32 | -2.65 | 4.73±0.39 | -5.43 | 4.84±0.27 | -3.22 |
|  | 0.2 | 0.21±0.01 | 5.32 | 0.19±0.01 | -8.12 | 0.21±0.02 | 7.42 | 0.20±0.00 | 3.98 |
| Methyl gallate | 100 | 97.35±6.23 | -2.66 | 95.38±8.35 | -4.62 | 93.28±7.32 | -6.72 | 98.42±3.52 | -1.58 |
|  | 10 | 10.38±0.93 | 3.78 | 9.52±0.87 | -4.83 | 10.24±0.92 | 2.43 | 10.32±0.85 | 0.32 |
|  | 0.4 | 0.37±0.03 | -7.52 | 0.37±0.03 | -0.15 | 0.38±0.03 | -5.22 | 0.42±0.03 | 5.12 |

**Table S6**. Main pharmacokinetic parameters of the 23 compounds from RB in rat plasma after oral administration of the single extract (RB) and the mixed extracts (RB-RPA). All data were expressed as mean ± SD, (*n* = 7).

| Compounds | Group | Parameters | | | | |
| --- | --- | --- | --- | --- | --- | --- |
|  |  | *t*_max_ /h | *t*_1/2_ /h | *C*_max_ /ng·mL^−1^ | AUC_0-_*_t_* /ng·mL^−1^·h | AUC_0-∞_ /ng·mL^−1^·h |
| Saikosaponin A | RB | 0.54±0.10 | 2.13±0.43 | 68.37±16.95 | 101.52± 38.54 | 103.55±38.59 |
|  | RB-RPA | 0.63±0.21 | 3.55±0.57 | 46.47±12.41 | 170.31±48.21 | 175.55±45.92 |
| Saikosaponin D | RB | 0.50±0.16 | 2.01±0.34 | 42.84±11.53 | 67.73±18.62 | 69.92±17.38 |
|  | RB-RPA | 0.87±0.38 | 2.98±0.89 | 26.57±6.99 | 99.98±31.48 | 103.56±33.67 |
| Saikosaponin C | RB | 0.50±0.16 | 2.07±1.12 | 31.12±11.08 | 38.82±9.84 | 42.15±8.73 |
|  | RB-RPA | 0.66±0.30 | 2.53± 1.54 | 20.63±5.65 | 51.33±16.19 | 53.67± 16.16 |
| Saikosaponin B_2_ | RB | 0.67±0.13 | 2.70±1.52 | 22.33±2.99 | 65.14±19.35 | 67.92±19.39 |
|  | RB-RPA | 0.83±0.20 | 2.89±1.14 | 28.19±5.50 | 98.63±18.36 | 104.39±23.28 |
| Acetyl-saikosaponin A | RB | 0.46±0.19 | 3.50±0.74 | 29.39±11.24 | 51.16±11.83 | 57.19± 10.74 |
|  | RB-RPA | 0.54±0.19 | 4.38±1.27 | 27.55±9.89 | 62.08±16.91 | 67.17±16.28 |
| Acetyl-saikosaponin B_2_ | RB | 0.42±0.13 | 3.81±1.29 | 14.82±4.41 | 16.86±4.34 | 19.75±3.88 |
|  | RB-RPA | 0.42±0.13 | 3.18±1.01 | 12.94±6.29 | 23.18±12.77 | 26.03± 13.58 |
| Acetyl-saikosaponin D | RB | 0.50±0.27 | 2.68±0.71 | 19.20±6.70 | 23.58±6.77 | 25.54±7.23 |
|  | RB-RPA | 0.50±0.27 | 4.39±1.55 | 20.31±12.42 | 38.05±21.32 | 43.25±27.08 |
| Prosaikogenin F | RB | 1.54±0.78 | 2.91±0.84 | 14.68±6.91 | 54.21±25.01 | 57.97±25.49 |
|  | RB-RPA | 1.62±0.74 | 3.46±0.55 | 18.36±8.50 | 71.62±34.91 | 77.58±36.46 |
| Prosaikogenin D | RB | 0.83±0.52  1.75±1.02 | 3.38±1.75 | 7.47±3.88  6.32±3.38 | 27.23±10.38 | 31.50±8.81 |
|  | RB-RPA | 0.71±0.40  1.86±0.92 | 2.98±1.07 | 8.53±4.68  8.42±4.54 | 32.79±15.58 | 34.59±14.83 |
| Prosaikogenin G | RB | 0.79±0.10 | 1.48±0.50 | 5.81±2.73 | 10.10±4.96 | 11.33±5.65 |
|  | RB-RPA | 0.75±0.16 | 1.48±0.63 | 6.08±3.17 | 11.50±5.88 | 12.94±6.57 |
| Saikogenin E | RB | 0.75±0.16 | 3.40±1.47 | 12.31±5.03 | 34.53±9.88 | 38.46±9.92 |
|  | RB-RPA | 0.79±0.10 | 3.99±2.19 | 13.41±6.28 | 40.24±15.68 | 45.24±14.83 |
| Saikogenin F | RB | 1.04±0.25 | 4.01±1.88 | 30.25±13.93 | 156.14±83.93 | 159.01±82.32 |
|  | RB-RPA | 1.17±0.26 | 6.80±2.11 | 34.09±17.25 | 301.08±199.77 | 347.02±174.43 |
| Saikogenin D | RB | 1.17±0.26 | 5.10±1.21 | 16.51±8.25 | 81.39±36.37 | 85.33±40.21 |
|  | RB-RPA | 0.92±0.20 | 5.78±2.37 | 19.75±10.49 | 131.78±59.75 | 141.32±65.28 |
| Saikogenin G | RB | 0.75±0.00 | 3.96±1.59 | 11.26±4.74 | 50.01±16.60 | 57.20±17.81 |
|  | RB-RPA | 0.71±0.10 | 6.53±2.21 | 12.67±4.42 | 88.79±41.87 | 99.59±41.70 |
| Hydroxy-saikogenin F | RB | 2.75±0.61 | 5.29±2.00 | 34.43±15.19 | 237.85±117.07 | 253.83±125.27 |
|  | RB-RPA | 3.08±1.11 | 5.07±2.65 | 37.67±18.73 | 284.07±151.66 | 299.39±151.57 |
| Dihydroxyl-dehydrogenation- saikogenin F | RB | 5.50±1.23 | 8.08±1.96 | 41.56±21.39 | 565.88±297.32 | 669.73±348.43 |
|  | RB-RPA | 4.67±0.82 | 9.89±4.62 | 53.36±28.16 | 770.57±432.52 | 1188.66±456.66 |
| Hydroxyl-dehydrogenation-saikogenin F | RB | 5.17±1.60 | 8.50±1.65 | 25.74±13.02 | 351.13±169.69 | 430.07±234.73 |
|  | RB-RPA | 4.67±0.82 | 8.58±1.88 | 31.81±13.51 | 454.10±194.14 | 588.75±229.02 |
| Dihydroxyl-saikogenin F | RB | 3.33±0.82 | 4.23±0.54 | 49.49±23.51 | 359.11±170.82 | 366.41±173.11 |
|  | RB-RPA | 3.00±0.00 | 4.12±1.37 | 51.59±26.78 | 427.20±192.43 | 437.57±195.32 |
| Trihydroxyl-dehydrogenation-saikogenin F | RB | 4.67±0.82 | 3.91±1.45 | 5.75±2.17 | 35.93±8.86 | 42.63±8.82 |
|  | RB-RPA | 4.67±0.82 | 3.33±1.09 | 7.37±2.58 | 47.58±19.85 | 54.43±21.64 |
| Hydroxy-saikogenin E | RB | 3.33±0.82 | 3.46±0.90 | 9.87±4.21 | 49.547±21.50 | 54.58±21.01 |
|  | RB-RPA | 2.75±0.61 | 3.05±0.40 | 8.91±4.37 | 53.19±25.97 | 58.61±29.74 |
| Dihydroxyl-dehydrogenation- saikogenin E | RB | 5.67±1.97 | 5.83±0.83 | 20.54±9.91 | 203.22±104.23 | 221.70±120.78 |
|  | RB-RPA | 4.42±1.43 | 9.33±1.75 | 25.39±12.57 | 348.34±191.81 | 467.14±231.86 |
| Hydroxyl-dehydrogenation- saikogenin E | RB | 5.50±1.23 | 3.03±0.91 | 6.56±3.25 | 39.92±18.64 | 43.64±18.60 |
|  | RB-RPA | 4.50±1.97 | 3.44±0.97 | 8.85±3.18 | 57.72±23.92 | 62.20±25.92 |
| Dihydroxyl-saikogenin E | RB | 2.75±0.61 | 2.74±0.37 | 10.47±4.55 | 60.01±18.25 | 64.15±30.19 |
|  | RB-RPA | 2.75±0.61 | 3.41±1.47 | 12.36±6.22 | 73.89±34.57 | 83.79±35.53 |

**Table S7**. Main pharmacokinetic parameters of the 15 compounds from RPA in rat plasma after oral administration of the single extract (RPA) and the mixed extracts (RB-RPA). All data were expressed as mean ± SD, (*n* = 7).

| Compounds | Group | Parameters | | | | | | | | |
| --- | --- | --- | --- | --- | --- | --- | --- | --- | --- | --- |
|  |  | *t*_max_ /h | | *t*_1/2_ /h | | *C*_max_ /ng·mL^−1^ | | AUC_0-_*_t_* /ng·mL^−1^·h | | AUC_0-∞_ /ng·mL^−1^·h |
| Paeoniflorin | RPA | 0.50±0.16 | | 2.71±0.72 | | 1776.42±513.40 | | 4255.05±868.84 | | 4264.82±873.40 |
|  | RB-RPA | 0.33±0.13 | | 1.96±0.37 | | 2932.12±385.77 | | 7165.68±1143.59 | | 7167.91±1144.02 |
| Albiflorin | RPA | 1.25±0.89 | | 3.19±0.58 | | 271.50±63.29 | | 1202.69± 302.31 | | 1210.33± 303.04 |
|  | RB-RPA | 0.71±0.25 | | 3.46±0.56 | | 365.94±41.75 | | 1834.06±191.89 | | 1849.80±396.92 |
| Oxypaeoniflorin | RPA | 0.71±0.19 | | 2.81±0.72 | | 21.62±2.97 | | 74.40± 21.91 | | 79.59± 25.23 |
|  | RB-RPA | 0.58±0.13 | | 2.87±0.71 | | 26.64±7.12 | | 93.27± 25.68 | | 99.49± 30.27 |
| Methyl gallate | RPA | 0.58±0.13 | | 4.11±2.31 | | 48.56±9.47 | | 140.81±61.26 | | 144.03±59.06 |
|  | RB-RPA | 0.50±0.16 | | 4.77±3.32 | | 57.65±7.34 | | 186.61±42.39 | | 212.56± 40.47 |
| Desbenzoylpaeoniflorin | RPA | 0.75±0.16 | | 2.72±1.32 | | 8.76±4.90 | | 18.66±9.99 | | 20.34±9.44 |
|  | RB-RPA | 0.58±0.13 | | 2.17±0.95 | | 10.12±5.32 | | 27.39±15.84 | | 28.66±16.13 |
| Methylgallic acid glucuronide | RPA | 6.33±2.58 | | 11.72±4.53 | | 85.28±24.44 | | 1240.17±313.17 | | 1723.01±554.35 |
|  | RB-RPA | 7.00±1.55 | | 11.06±6.32 | | 105.82±34.86 | | 1437.33±310.18 | | 2082.54±511.34 |
| Methylgallic acid sulfate | RPA | 3.08±1.11 | | 3.03±1.31 | | 17.76±5.10 | | 71.89±26.20 | | 76.36±28.87 |
|  | RB-RPA | 3.08±1.11 | | 5.22±1.95 | | 14.45±5.53 | | 119.34±52.47 | | 126.46±50.77 |
| 3,4-di-*O*-methyl gallic acid sulfate | RPA | 1.46±0.81 | | 6.80±2.59 | | 8.32±2.81 | | 85.32± 31.73 | | 97.99±29.10 |
|  | RB-RPA | 1.33±0.82 | | 5.07±1.83 | | 7.91±4.09 | | 50.23± 20.16 | | 59.59±23.85 |
| Pyrogallol glucuronide | RPA | | 2.67±1.85 | | 6.16±2.02 | | 34.21±4.94 | | 262.73±101.68 | 336.41±159.76 |
|  | RB-RPA | | 2.92±1.43 | | 10.49±2.78 | | 47.97±13.98 | | 643.56±348.44 | 825.47±393.90 |
| Pyrogallol sulfate | RPA | | 2.50±0.78 | | 7.72±3.81 | | 18.42±5.81 | | 164.21±79.60 | 187.63±91.25 |
|  | RB-RPA | | 1.75±0.61 | | 7.54±3.63 | | 24.16±13.26 | | 217.62±126.82 | 257.66±175.61 |
| Methylpyrogallol sulfate | RPA | | 3.33±0.81 | | 6.70±1.28 | | 57.35±21.95 | | 579.49±257.63 | 638.90±286.06 |
|  | RB-RPA | | 4.25±2.28 | | 7.97±3.51 | | 63.18±20.78 | | 837.85±407.48 | 1042.62±632.06 |
| Methylpyrogallol glucuronide | RPA | | 4.33±1.03 | | 6.35±3.00 | | 23.02±10.52 | | 169.96±78.63 | 183.67±75.44 |
|  | RB-RPA | | 4.42±1.43 | | 8.15±4.29 | | 29.04±9.69 | | 304.97±128.19 | 363.84±125.30 |
| Paeonimetabolin I | RPA | | 0.75±0.00 | | 7.39±0.45 | | 658.61±349.75 | | 4864.64±2525.32 | 5467.27±2837.33 |
|  | RB-RPA | | 0.83±0.13 | | 7.11±2.01 | | 575.13±257.81 | | 4248.67±1970.10 | 4806.68±2129.42 |
| Paeonimetabolin II | RPA | | 1.04±0.37 | | 9.22±2.04 | | 850.12±292.63 | | 6694.27±2468.69 | 8022.12±2764.81 |
|  | RB-RPA | | 1.17±0.90 | | 9.30±2.76 | | 984.50±452.44 | | 9490.91±4705.90 | 13198.67±4477.34 |
| Paeonimetabolin I glucuronide isomer | RPA | | 1.08±0.20 | | 1.89±0.38 | | 23.3±6.10 | | 91.88±43.73 | 97.36±45.56 |
|  | RB-RPA | | 1.17±0.26 | | 2.10±0.28 | | 19.20±9.49 | | 74.56±33.42 | 79.46±34.34 |


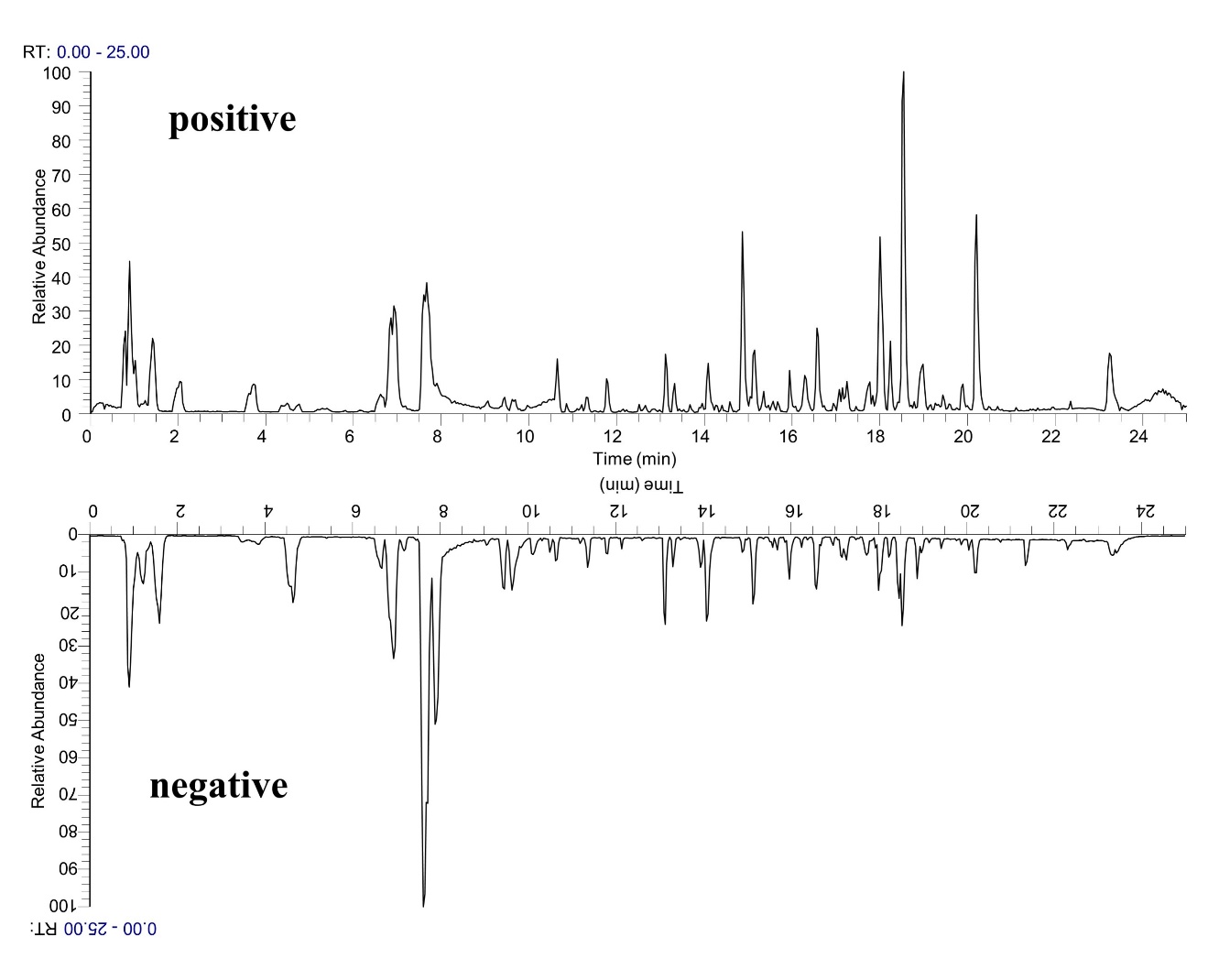


**Fig. S3**. UPLC-MS/MS base peak chromatograms of plasma samples after oral administration of RB-RPA

**Table S8**. Relative distance calculation between post-dose all time points metabolite profiles and pre-dose metabolite profile from the score plot of PCA with average value (x-axis and y-axis) of all samples. Values are presented as mean± SD, (*n* = 6).

| pre-dose time point (0 h) | | Each time points after administration | Distance |
| --- | --- | --- | --- |
| x-Axis (mean) | y-Axis (mean) |  |  |
| 6.23 | 19.88 | 0.083 h | 11.42±0.92 |
|  |  | 0.25 h | 17.62±0.71 |
|  |  | 0.5 h | 20.75±0.63 |
|  |  | 0.75 h | 19.89±0.68 |
|  |  | 1 h  1.5 h | 26.52±0.74  29.45±0.78 |
|  |  | 3 h  5 h  8 h  12 h  24 h | 34.66±0.89  29.81±1.31  27.71±0.98  26.41±1.52  14.15±0.86 |

**Table S9.** List of the altered endogenous metabolites in response to RB-RPA intervention, and the data were normalized by IS (glycyrrhizin). Values are presented as mean± SD, (*n* = 6).

| No. | Metabolites | 0 h | 0.083 h | | 0.25 h | | 0.5 h | 0.75 h | 1 h | 1.5 h | 3 h | 5 h | 8 h | 12 h | 24 h | |
| --- | --- | --- | --- | --- | --- | --- | --- | --- | --- | --- | --- | --- | --- | --- | --- | --- |
| 1 | DL-Ornithine | 0.158±0.023 | 0.118±0.044 | 0.198±0.053 | | 0.186±0.061 | | 0.213±0.077 | 0.241±0.064 | 0.233±0.074 | 0.251±0.089 | 0.271±0.091 | 0.223±0.078 | 0.143±0.088 | | 0.166±0.053 |
| 2 | DL-Histidine | 0.252±0.032 | 0.291±0.036 | 0.277±0.064 | | 0.312±0.088 | | 0.201±0.092 | 0.365±0.076 | 0.292±0.043 | 0.321±0.071 | 0.387±0.085 | 0.282±0.065 | 0.221±0.083 | | 0.232±0.095 |
| 3 | Choline | 2.392±0.143 | 2.434±0.264 | 2.954±0.942 | | 1.432±0.743 | | 1.954±0.533 | 3.481±0.768 | 3.734±1.059 | 4.066±1.235 | 3.893±1.188 | 3.622±1.365 | 3.283±0.845 | | 2.717±0.974 |
| 4 | gamma-Aminobutyric acid | 0.064±0.018 | 0.057±0.016 | 0.071±0.022 | | 0.078±0.021 | | 0.084±0.029 | 0.091±0.033 | 0.121±0.038 | 0.109±0.031 | 0.092±0.034 | 0.112±0.044 | 0.074±0.024 | | 0.055±0.020 |
| 5 | L-Glutamic acid | 0.205±0.021 | 0.242±0.033 | 0.165±0.077 | | 0.294±0.038 | | 0.223±0.065 | 0.399±0.051 | 0.378±0.072 | 0.449±0.051 | 0.456±0.066 | 0.476±0.083 | 0.418±0.079 | | 0.375±0.067 |
| 6 | Valine | 5.557±1.824 | 5.508±1.792 | 5.834±1.943 | | 6.285±1.921 | | 7.021±2.191 | 8.651±2.624 | 9.214±2.415 | 9.008±2.149 | 8.771±3.012 | 7.363±3.131 | 6.411±2.341 | | 5.913±2.041 |
| 7 | DL-Glutamine | 0.106±0.018 | 0.144±0.023 | 0.111±0.010 | | 0.165±0.071 | | 0.074±0.022 | 0.061±0.008 | 0.066±0.012 | 0.048±0.009 | 0.131±0.097 | 0.033±0.006 | 0.061±0.009 | | 0.085±0.012 |
| 8 | alpha-Ketoglutaric acid | 0.177±0.033 | 0.182±0.041 | 0.204±0.039 | | 0.288±0.044 | | 0.331±0.025 | 0.387±0.044 | 0.433±0.049 | 0.398±0.032 | 0.353±0.025 | 0.319±0.030 | 0.234±0.041 | | 0.199±0.036 |
| 9 | L-(-)-Asparagine | 0.063±0.007 | 0.049±0.011 | 0.041±0.008 | | 0.068±0.014 | | 0.075±0.012 | 0.085±0.017 | 0.051±0.014 | 0.077±0.011 | 0.058±0.008 | 0.031±0.005 | 0.064±0.006 | | 0.059±0.010 |
| 10 | Citric acid | 0.104±0.011 | 0.097±0.008 | 0.143±0.052 | | 0.088±0.023 | | 0.079±0.018 | 0.066±0.027 | 0.071±0.010 | 0.050±0.009 | 0.047±0.006 | 0.069±0.011 | 0.121±0.032 | | 0.087±0.029 |
| 11 | L-Tyrosine | 5.902±1.639 | 5.212±1.453 | 4.029±1.582 | | 3.206±1.734 | | 6.349±1.349 | 5.413±1.022 | 5.559±1.183 | 2.645±0.892 | 4.952±1.005 | 5.214±1.325 | 6.525±2.109 | | 5.311±1.444 |
| 12 | Itaconic acid | 0.008±0.001 | 0.011±0.003 | 0.013±0.003 | | 0.015±0.004 | | 0.019±0.005 | 0.024±0.005 | 0.017±0.007 | 0.020±0.007 | 0.016±0.009 | 0.014±0.006 | 0.010±0.003 | | 0.012±0.004 |
| 13 | Picolinic acid | 0.021±0.007 | 0.023±0.006 | 0.031±0.008 | | 0.039±0.011 | | 0.046±0.014 | 0.041±0.022 | 0.033±0.009 | 0.043±0.013 | 0.031±0.010 | 0.023±0.009 | 0.016±0.007 | | 0.019±0.008 |
| 14 | DL-Tryptophan | 0.012±0.001 | 0.018±0.006 | 0.012±0.005 | | 0.009±0.002 | | 0.006±0.003 | 0.010±0.005 | 0.006±0.002 | 0.009±0.003 | 0.008±0.001 | 0.012±0.004 | 0.008±0.002 | | 0.014±0.005 |
| 15 | N-Acetyl-L-leucine | 0.018±0.003 | 0.020±0.004 | 0.011±0.006 | | 0.019±0.003 | | 0.021±0.002 | 0.017±0.008 | 0.015±0.007 | 0.024±0.002 | 0.022±0.003 | 0.021±0.03 | 0.016±0.09 | | 0.020±0.004 |
| 16 | L-Glutathione oxidized | 0.061±0.008 | 0.055±0.012 | 0.048±0.015 | | 0.033±0.011 | | 0.021±0.008 | 0.027±0.009 | 0.033±0.007 | 0.036±0.009 | 0.048±0.011 | 0.051±0.013 | 0.053±0.021 | | 0.063±0.019 |
| 17 | Thymidine 5'-monophosphate | 0.143±0.022 | 0.121±0.034 | 0.130±0.028 | | 0.157±0.042 | | 0.128±0.036 | 0.119±0.042 | 0.123±0.039 | 0.114±0.027 | 0.126±0.033 | 0.119±0.029 | 0.133±0.042 | | 0.139±0.038 |
| 18 | Taurochenodeoxycholic acid | 0.011±0.003 | 0.013±0005 | 0.015±0.06 | | 0.012±0.005 | | 0.009±0.002 | 0.010±0.004 | 0.008±0.001 | 0.007±0.001 | 0.009±0.003 | 0.011±0.004 | 0.008±0.003 | | 0.010±0.002 |
| 19 | Cholic acid | 0.043±0.009 | 0.040±0.007 | 0.037±0.011 | | 0.031±0.010 | | 0.028±0.007 | 0.021±0.006 | 0.016±0.003 | 0.019±0.002 | 0.025±0.005 | 0.029±0.007 | 0.037±0.009 | | 0.041±0.012 |
| 20 | α-Linolenic acid | 0.039±0.005 | 0.031±0.011 | 0.058±0.013 | | 0.062±0.012 | | 0.087±0.013 | 0.058±0.020 | 0.056±0.011 | 0.049±0.011 | 0.047±0.015 | 0.042±0.015 | 0.036±0.012 | | 0.038±0.010 |
| 21 | LysoPC(18:3) | 0.123±0.032 | 0.126±0.029 | 0.113±0.041 | | 0.108±0.036 | | 0.103±0.049 | 0.095±0.015 | 0.107±0.021 | 0.093±0.035 | 0.087±0.022 | 0.097±0.017 | 0.118±0.019 | | 0.114±0.020 |
